# Comprehensive analysis of extensive drug-resistant *Salmonella* Typhi in Gujarat region, India: genomic findings and prospective alternative therapy

**DOI:** 10.1128/spectrum.02540-24

**Published:** 2025-05-27

**Authors:** Sadanand Dangari Akshay, Heli Upadhyaya, Nitin Shukla, Rohit Bhattacharjee, Sunilkumar Das, Urmi Vyas, Priyank Chavda, Nimesh Patel, Dixsha Jamkhandi, Pritesh Sabara, Neeta Khandelwal, Sumeeta Soni, Kamlesh Upadhyaya, Jayesh Katira, Geeti Maheshwari, Madhvi Joshi, Devarshi Gajjar, Chaitanya Joshi

**Affiliations:** 1 Department of Science & Technology, Gujarat Biotechnology Research Centre, Government of Gujarat 124277 , Gandhinagar, Gujarat, India; 2 Department of Microbiology and Biotechnology Centre, Faculty of Science, The Maharaja Sayajirao University of Baroda 226962, , Vadodara, Gujarat, India; 3 B.J. Medical College, New Civil Hospital, Ahmedabad, Gujarat, India; 4 Commissionerate of Health, Department of Health and Family Welfare, Government of Gujarat 163890 , Gandhinagar, Gujarat, India; 5 Toprani Advanced Lab Systems, Vadodara, Gujarat, India; Institut National de Santé Publique du Québec, Sainte-Anne-de-Bellevue, Québec, Canada

**Keywords:** antibiotic resistance, alternative therapy, *S*. Typhi, PMQR, QRDR

## Abstract

Antimicrobial resistance (AMR) in *Salmonella* Typhi (*S*. Typhi) is a serious health issue that can complicate typhoid fever treatment and increase fatality rates. The National Centre for Disease Control, Government of India, established a National Antimicrobial Surveillance Network to detect AMR in high-priority infections, including *S*. Typhi throughout geographical areas of India, and understand AMR dissemination and possible treatment options. The Gujarat State Biotechnology Mission, Government of Gujarat, India, has funded the “Network Program on Antimicrobial Resistance, Superbugs, and One Health” to track antibiotic resistance patterns in the Gujarat region. Given the enormity of the AMR problem in *S*. Typhi, in Gujarat, this study evaluated the antibiotic resistance patterns of *S*. Typhi isolates from the Ahmedabad and Vadodara regions of Gujarat, India, were used to determine the mechanism underlying antibiotic resistance and developed combination treatments to combat the extensive drug-resistant isolates. We collected 122 *Salmonella* isolates periodically (2022–2023) in the Ahmedabad and Vadodara regions of Gujarat, India. The antibiotic susceptibility for 122 *S*. Typhi isolates was prepared using the disc diffusion method and VITEK II compact system. Whole-genome sequencing and *in-silico* analysis were performed to identify potential antibiotic resistance genes (ARGs); in addition, combination antibiotic therapies were investigated, and resistance patterns were predicted for key antibiotics used in the treatment of typhoid fever. The study discovered notable levels of antibiotic resistance, specifically to cefuroxime (97.54%), doxycycline (97.54%), ceftriaxone (95.90%), cotrimoxazole (95.90%), ampicillin/sulbactam (95.90%), cefotaxime (94.26%), and ciprofloxacin (94.26%). Genetic analysis revealed the presence of *aph(3'')-Ib* (40.16%), *aph(6)-Id* (95.08%), *bla*_CTX-M_ (89.39%), *dfrA14* (68.85%), plasmid-mediated quinolone resistance (PMQR), and the *qnrS1* gene (94.26%). IncFIB(K) and IncFIB(pHCM2) were detected in 96.71% of *S*. Typhi isolates. Point mutations in the QRDR region of *gyrA* identified three distinct modifications: Ser83Phen (91.80%), Ser83Leu (1.64%), and Ser83Tyr (0.82%). Three genes exhibited point mutations: *gyrB* (100%), Ile253Val, and *parC* (6.56%) multiple mutations, and the efflux gene *mdtK* (100%) Thr224Lys. By evaluating phenotype and genotype, the tests showed an average concordance of 91.69% and a discordance of 8.31%. The study revealed that 97.54% of isolates belonged to the H58 haplotype lineage II (ST1: 4.3.1.2), which is associated with high resistance to cephalosporins and fluoroquinolones in Southeast Asia. The combination therapy of *β*-lactam/*β*-lactamase inhibitors (BL/BLI) demonstrated good outcomes, with low MICs (<0.5 µg/mL) of ceftazidime/tazobactam, cefixime/tazobactam, and ceftriaxone/tazobactam effective against highly resistant *S*. Typhi. The emergence of MDR–XDR *S*. Typhi, attributed to mutations in *gyrA*, parC, and the H58 lineage, highlights widespread resistance. According to our research, a potential strategy against XDR *S*. Typhi is a combination treatment with BL/BLI.

**IMPORTANCE:**

One of the first studies to assess the antimicrobial resistance patterns of *S*. Typhi isolates from the Ahmedabad and Vadodara regions; this particular investigation provides vital information on the occurrence of key resistance genes and mechanisms. This study has significantly contributed by finding the β-lactam/β-lactamase inhibitor combination therapy as an appropriate treatment choice for XDR, which can potentially use *S*. Typhi on a larger scale. Additionally, it provides the antibiotic resistance prediction of key antibiotics used for treating typhoidal fever. The outcomes of this investigation highlight the urgent need to address the surge of XDR *S*. Typhi in high-burden regions of Gujarat. Our study highlights the possibility of rapid dissemination of antibiotic resistance due to chromosomal point mutations and plasmid-mediated gene transfer. The effectiveness of β-lactam/β-lactamase inhibitors in handling XDR *S*. Typhi suggests a plausible strategy for treatment that may be included among clinical guidelines.

## INTRODUCTION

Antibiotic resistance in *Salmonella* has become the most pressing issue globally. A World Health Organization report stated that antibiotic resistance causes 700,000 fatalities annually (1). Similar remarks by the Interagency Coordination Group (IACG) (IACG 2019) anticipate that multi-drug resistance (MDR) fatalities worldwide will rise to 10 million by 2030 (2). The decreasing pace of new antibiotic development, the increase in pan drug resistance (PDR), and the onset of extensive drug resistance (XDR) pose serious risks to the hospital environment. The 2019 antimicrobial threat report from the Centers for Disease Control and Prevention (CDC) predicted that *Salmonella enterica* serotype Typhi (*S*. Typhi), causes 11–21 million infections every year. *S*. Typhi is the agent that causes typhoid fever, a severe enteric disease that spreads through contaminated food and water (3, 4). In the early 21^st^ century, the most common antibiotics used to treat typhoid fever were azithromycin, ceftriaxone, and ciprofloxacin. Because of severe toxicity, fourth-generation fluoroquinolones (FQs) (Gatifloxacin) were banned by the Indian government in 2011, followed by the restricted use of moxifloxacin in the treatment of typhoid fever (5). The uncontrolled use of antibiotics for common diseases, particularly non-bacterial infections, has resulted in the emergence of MDR, and this has considerably raised the mortality rate of typhoid fever (5).

Antibiotic resistance can occur through several factors, such as drug inactivation, target modification, antibiotic efflux, decreased membrane permeability, plasmid acquisition, or chromosome mutation (6). One of the main contributors to antibiotic resistance in *S*. Typhi is a form of horizontal gene transfer (HGT) via mobile genetic elements that consist of plasmids, transposons, and phages (7). Plasmids are prominently featured as agents of HGT involved in the transmission of antibiotic resistance genes (ARGs) in *S*. Typhi. IncFIB(K) is one of the primary plasmids in *Salmonella* for the spread of ARGs; also, close interconnection with Enterobacterales has created the chance of spreading MDR in a hospital environment. Previous research suggests that if compensatory evolution offsets the expense of plasmid transport, H58 lineage II might acquire MDR plasmids from Enterobacterales (8). Over the last two decades, the dominant *S*. Typhi haplotype, often known as H58, has spread globally and is commonly designated by MDR. This specific haplotype is now prevalent throughout Southeast Asia and sub-Saharan Africa. Several localized typhoid outbreaks have been attributed to various sub-lineages from H58 (9). Plasmids can carry a variety of ARGs, including *bla*_CTX-M_, *bla*_TEM_, *bla*_VIM_, *bla*_NDM_, *qnrS*, *aac*, *sul*, *dfr*, *mcr*, *cat*, *tet*, and others, which limit the susceptibility of crucial last-line antibiotics against typhoid fever (6, 9). As a consequence, the treatment options for typhoid fever are limited, which may increase mortality rates and hinder economic progress, particularly among developing and underdeveloped countries. Considering the seriousness of typhoid fever and its enormous potential for a rise in mortality due to the development of MDR to XDR, this study is a humble attempt to recapitulate our findings by investigating the spread of antibiotic resistance through HGT and mutations in straight genes, as well as the combination of antibiotics for increasing susceptibility to *S*. Typhi.

## RESULTS

### General demographics and *Salmonella* Spp. identification

**Fig 1:**
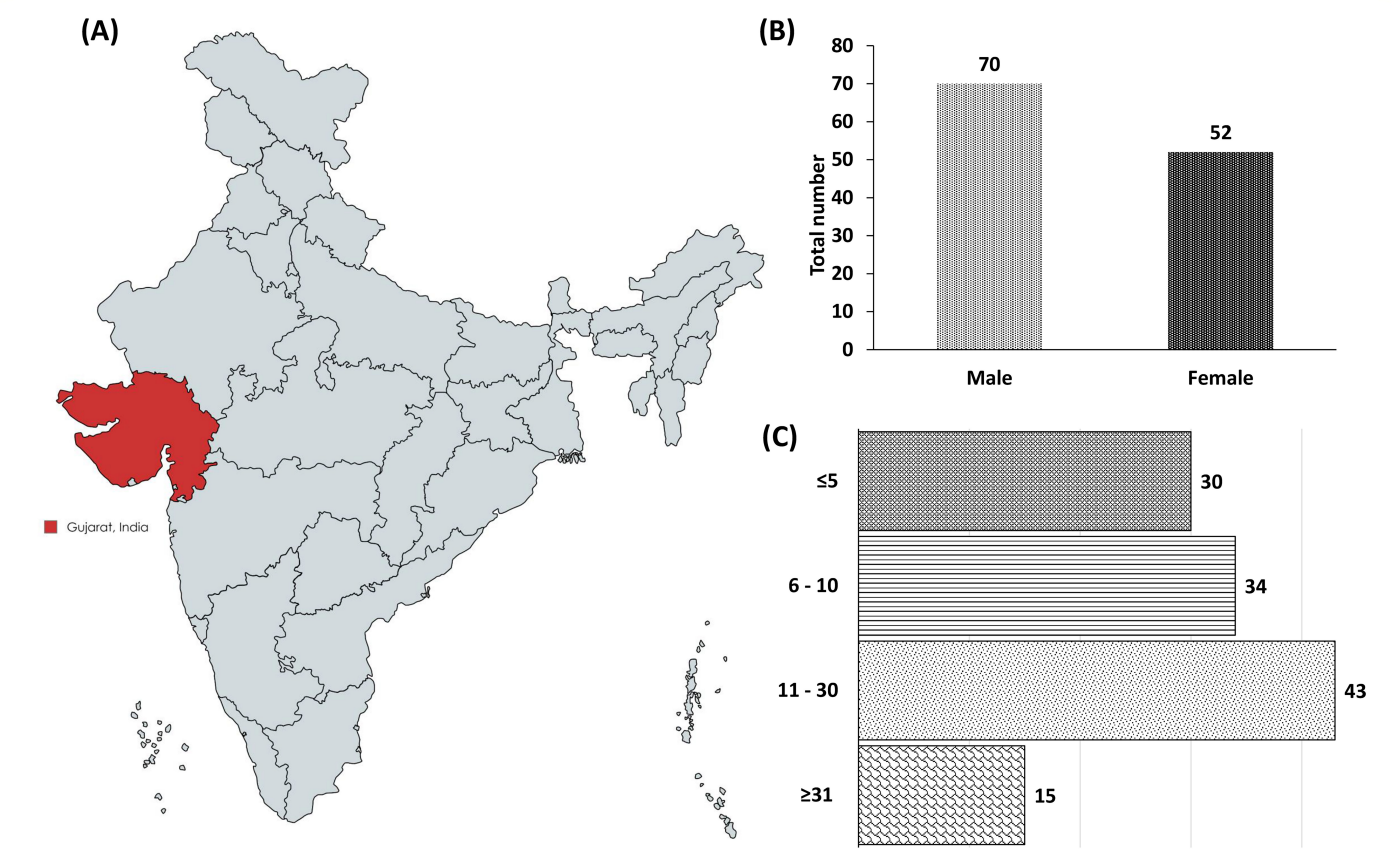
Demographic distribution of the *S*. Typhi isolates collected during the study period. (**A**) Isolate collection graphic depiction. The map was created using MapChart (https://www.mapchart.net/). (**B**) Number of *S*. Typhi isolated from males and females (**C**) The distribution of *S*. Typhi across the age groups.

Of the 122 *S*. Typhi isolates, 70 (57.38%) were isolated from male patients and 52 (42.62%) from female patients (Fig. 1A and B). In addition, children under the age of ≤5 account for 30/122 (24.60%). Another age group that includes 6–10 accounts for 34/122 (27.86%), 11–30 accounts for 43/122 (35.24%), and ≥31 accounts for 15/122 (12.30%) (Fig. 1C). All the 122 gram-negative isolates were identified in MALDI-TOF as *Salmonella* spp., with a minimum (log)score of <1.71 and higher log(score) of >2. A detailed spectrum profile is provided in File S1.

### Antibiotic sensitivity in *Salmonella*

The study employed 28 therapeutically relevant antibiotics as the possible treatments for typhoidal fever (3, 4, 6, 8, 10). The highest resistance was observed for pefloxacin (99.18%), cefuroxime (97.54%), tetracycline (97.54%), doxycycline (97.54%), cefuroxime-axetil (96.72%), ceftriaxone (95.90%), cefazolin (95.08%), co-trimoxazole (95.90%), ampicillin/sulbactam (95.90%), cefotaxime (94.26%), cefixime (94.26%), ciprofloxacin (94.2%), and gentamicin (94.26%). The lowest resistance was observed for chloramphenicol (9.01%), ertapenem (7.37%), cefoperazone-sulbactam (3.2%), colistin (1.63%), amoxicillin/clavulanic acid (1.63%), tigecycline (0.81%), and fosfomycin (0.81%). A detailed result is provided in Table S2.

### Whole genome sequencing (WGS) of *S*. Typhi

A total of 12,182 contigs were generated, with an average of 99.85 per genome from 122 *S*. Typhi, with a total size of 602.82 million data reads in assemblies. The sequence had an average G  +  C content of 51.99%, resembling other isolates that corroborate with *Salmonella*. The raw WGS data were deposited in the Indian Biological Data Centre (IBDC). The details of the isolates, including genome assembly statistics, are summarized in Table S3.

### Serotyping and *in silico* MLST typing of *Salmonella*

**Fig 2:**
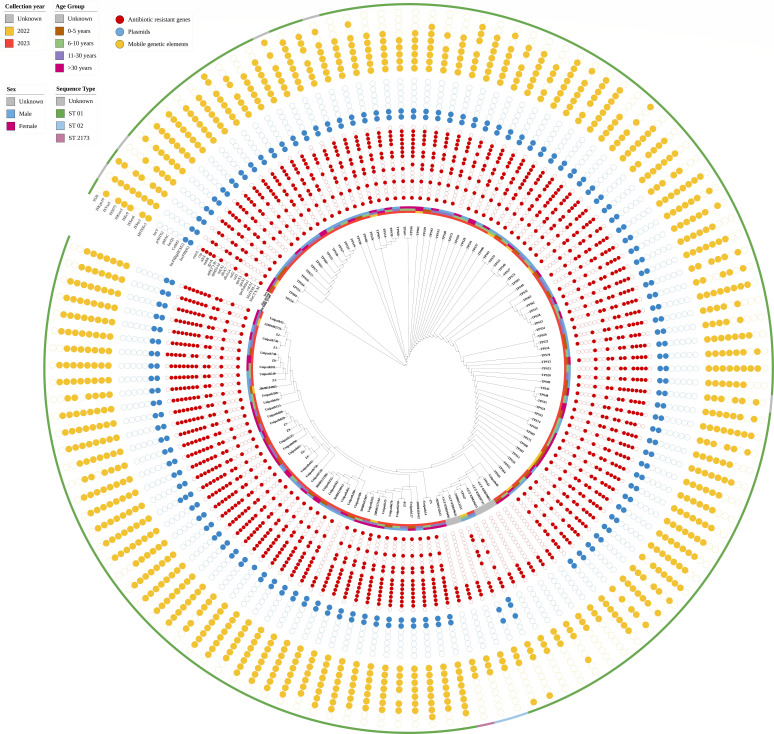
Circular phylogenetic tree of 122 *S.* Typhi sequence. The outer circle (golden yellow) indicates the distribution of mobile genetic elements. The inner circle with/without Cornflower blue color represents the distribution of the plasmid. The inner circle with crimson red color represents the distribution of the antibiotic resistance gene. Inner blocks represent sex, age, and collection year.

The 53 genes encoding bacterial ribosome protein components (*rps* genes) were employed for initial species-level identification using an *in-silico* rMLST (ribosomal multi-locus sequence typing) technique that combined microbial taxonomy and typing. Among the 122 detected *Salmonella enterica* subsp. *enterica* isolates, three distinct *Salmonella* serovars were discovered, with the majority of them comprising *S*. Typhi (120/122) and two strains of, *Salmonella* Ndolo (1/122) and *Salmonella* Gallinarum (1/122). Sequence Types (STs) were generated by employing seven loci, namely *sucA , aroC*, , *dnaN*, *thrA*, *purE*, *hemD,* and *hisD,* based on data from the MLST database of *Salmonella enterica* (11). Among the 122 *Salmonella* isolates, two distinct ST types have been determined: 120 isolates (98.36%) belonged to ST1 (*S*. Typhi), one isolate (0.82%) belonged to ST 2173 (*S*. Typhi), and one isolate (0.82%) did not display the serotype. The clonal complex-13 comprised the entire 122 STs, and three distinct genotypes were determined: 4.3.1.2 (ST1; *S*. Typhi) included 119 isolates (97.54%), whereas 4.3.1.1 (ST1; *S*. Typhi) included two isolates (1.64%) and 2.2.1 (ST1; *S*. Typhi) included one isolate (0.82%). Three distinct genotypes (2.2.1, 4.3.1.1, and 4.3.1.2) were identified in the genotype categorization based on the Wong et al. (12) framework, illustrating the varied population pattern in India. H58 lineage II (genotype 4.3.1.2) accounted for 97.54% of the isolates, whereas H58 lineage I (1.64%) included the remaining isolates of *S*. Typhi collected in Gujarat, India, during, July and August of 2023. Details of the isolate with genotypic classification are shown in Table 1 (Fig. 2).

**TABLE 1:** A comprehensive overview of the structural investigation of 122 isolates of *S*. Typhi

No.	Isolate	Serotype	ST type	Clonal complex	Antigenic profile	Plasmids present	Mobile genetic elements	Antibiotic resistance genes	Accession Number
20600104985	*Salmonella enterica*	Typhi	ST 1	13	9:d:-	IncFIB(K), IncFIB(pHCM2)	ISEc9, ISKpn19, ISSen6, ISVsa5, ISKox3, IS5075, ISSty2, MITEEc1	*qnrS1*, *bla_CTX-M_*, *sul2*, *dfrA14*, *tet(A*), *aph(6)-Id*, *aph(3'')-Ib*, *gyrA*, *mdtK*, *sdiA*, *crp*	INS0016998
20800127544	*Salmonella enterica*	Typhi	ST 1	13	9:d:-	IncFIB(K), IncFIB(pHCM2)	ISEc9, ISKpn19, ISSen6, ISVsa5, ISKox3, IS5075, ISSty2, MITEEc1	*qnrS1*, *bla_CTX-M_*, *sul2*, *dfrA14*, *tet(A*), *aph(6)-Id*, *aph(3'')-Ib*, *gyrA*, *mdtK*, *sdiA*, *crp*	INS0016999
21000113251	*Salmonella enterica*	Typhi	ST 1	13	9:d:-	IncFIB(pHCM2) IncQ1	IS26, ISSen6, ISSty2, MITEEc1	*catA1, qacEdelta1, sul1, bla_TEM-1_, sul2, mdtK, dfrA7, sdiA, crp*	INS0017000
21001002751	*Salmonella enterica*	Typhi	ST 1	13	9:d:-	IncFIB(K), IncFIB(pHCM2)	ISEc9, ISSen6, ISVsa5, ISKox3, IS5075, ISSty2, MITEEc1	*qnrS1*, *bla_CTX-M_*, *sul2*, *dfrA14*, *tet(A*), *aph(6)-Id*, *aph(3'')-Ib*, *gyrA*, *mdtK*, *sdiA*, *crp*	INS0017001
30300118412	*Salmonella enterica*	Typhi	ST 1	13	9:d:-	IncFIB(K), IncFIB(pHCM2)	ISEc9, ISKpn19, ISSen6, ISVsa5, ISKox3, IS5075, ISSty2, MITEEc1	*qnrS1*, *bla_CTX-M_*, *sul2*, *dfrA14*, *tet(A*), *aph(6)-Id*, *aph(3'')-Ib*, *gyrA*, *mdtK*, *sdiA*, *crp*	INS0017003
30300132615	*Salmonella enterica*	Typhi	ST 2173	13	9:d:-	^*a*^-	ISSen6, IS903, ISSty2, MITEEc1	*gyrA*, *mdtK*, *sdiA*, *crp*	INS0017004
30400119912	*Salmonella enterica*	Typhi	ST 1	13	9:d:-	IncFIB(K), IncFIB(pHCM2)	ISEc9, ISKpn19, ISSen6, ISVsa5, ISKox3, IS5075, ISSty2, MITEEc1	*qnrS1*, *bla_CTX-M_*, *sul2*, *dfrA14*, *tet(A*), *aph(6)-Id*, *aph(3'')-Ib*, *gyrA*, *mdtK*, *sdiA*, *crp*	INS0017005
30400107987	*Salmonella enterica*	Typhi	ST 1	13	9:d:-	IncFIB(K), IncFIB(pHCM2)	ISEc9, ISSen6, ISVsa5, ISKox3, IS5075, ISSty2, MITEEc1	*qnrS1, bla_CTX-M_, sul2, dfrA14, tet(A), aph(6)-Id, aph(3'')-Ib, gyrA, mdtK, sdiA, crp*	INS0017002
30600122390	*Salmonella enterica*	Typhi	ST 1	13	9:d:-	IncFIB(K)	ISEc9, ISKpn19, ISSen6, ISVsa5, ISKox3, IS5075, ISSty2, MITEEc1	*qnrS1, bla_CTX-M_, sul2, dfrA14, tet(A), aph(6)-Id, aph(3'')-Ib, gyrA, mdtK, sdiA, crp*	INS0017006
TPS01	*Salmonella enterica*	Typhi	ST 1	13	9:d:-	IncFIB(K), IncFIB(pHCM2)	MITEEc1, ISSty2, ISSen6, ISEc9, ISKox3, IS5075, ISVsa5	*qnrS1, bla_CTX-M_, sul2, dfrA14, tet(A), aph(6)-Id, gyrA, mdtK, sdiA*	INS0017007
TPS02	*Salmonella enterica*	Typhi	ST 1	13	9:d:-	IncFIB(K), IncFIB(pHCM2)	MITEEc1, ISSty2, ISSen6, ISEc9, ISKox3, IS5075, ISVsa5	*qnrS1, bla_CTX-M_, sul2, dfrA14, tet(A), aph(6)-Id, gyrA, mdtK, sdiA, crp*	INS0017017
TPS03	*Salmonella enterica*	Typhi	ST 1	13	9:d:-	IncFIB(K), IncFIB(pHCM2)	MITEEc1, ISSty2, ISSen6, ISEc9, ISKox3, IS5075	*qnrS1*, *bla_CTX-M_*, *sul2*, *bla_TEM-1,_ dfrA14*, *tet(A*), *aph(6)-Id*, *gyrA*, *mdtK*, *sdiA*, *crp*	INS0017028
TPS04	*Salmonella enterica*	Ndolo	----		9:d:1,5	IncFIB(K), IncFIB(pHCM2)	MITEEc1, ISSty2, ISSen6, ISEc9, ISKox3, IS5075, ISVsa5, ISKpn19	*bla_CTX-M_*, *sul2*, *dfrA14*, *tet(A*), *aph(6)-Id*, *mdtK*, *sdiA*	INS0017039
TPS05	*Salmonella enterica*	Typhi	ST 1	13	9:d:-	IncFIB(K), IncFIB(pHCM2)	MITEEc1, ISSty2, ISSen6, ISEc9, ISKox3, IS5075, ISVsa5	*qnrS1*, *bla_CTX-M_*, *sul2*, *dfrA14*, *tet(A*), *aph(6)-Id*, *gyrA*, *mdtK*, *sdiA*, *crp*	INS0017050
TPS06	*Salmonella enterica*	Typhi	ST 1	13	9:d:-	IncFIB(K), IncFIB(pHCM2)	MITEEc1, ISSty2, ISSen6, ISEc9, ISKox3, IS5075, ISKpn19	*qnrS1*, *bla_CTX-M_*, *sul2*, *dfrA14*, *tet(A*), *aph(6)-Id*, *aph(3'')-Ib*, *gyrA*, *mdtK*, *sdiA*, *crp*	INS0017061
TPS07	*Salmonella enterica*	Typhi	ST 1	13	9:d:-	IncFIB(K), IncFIB(pHCM2)	MITEEc1, ISSty2, ISSen6, ISEc9, ISKox3, IS5075, ISVsa5	*qnrS1*, *bla_CTX-M_*, *sul2*, *dfrA14*, *tet(A*), *aph(6)-Id*, *gyrA*, *mdtK*, *sdiA*, *crp*	INS0017072
TPS08	*Salmonella enterica*	Typhi	ST 1	13	9:d:-	IncFIB(K), IncFIB(pHCM2)	MITEEc1, ISSty2, ISSen6, ISEc9, ISKox3, IS5075, ISVsa5	*qnrS1*, *bla_CTX-M_*, *sul2*, *dfrA14*, *tet(A*), *aph(6)-Id*, *gyrA*, *mdtK*, *sdiA*, *crp*	INS0017079
TPS09	*Salmonella enterica*	Typhi	ST 1	13	9:d:-	IncFIB(K), IncFIB(pHCM2)	MITEEc1, ISSty2, ISSen6, ISKox3, IS5075, ISKpn19	*qnrS1*, *bla_CTX-M_*, *sul2*, *dfrA14*, *tet(A*), *aph(6)-Id*, *aph(3'')-Ib*, *gyrA*, *mdtK*, *sdiA*, *crp*	INS0017080
TPS10	*Salmonella enterica*	Typhi	ST 1	13	9:d:-	IncFIB(K), IncFIB(pHCM2)	MITEEc1, ISSty2, ISSen6, ISEc9, ISKox3, IS5075, ISKpn19	*qnrS1*, *bla_CTX-M_*, *sul2*, *dfrA14*, *tet(A*), *aph(6)-Id*, *gyrA*, *mdtK*, *sdiA*, *crp*	INS0017008
TPS11	*Salmonella enterica*	Typhi	ST 1	13	9:d:-	IncFIB(K), IncFIB(pHCM2)	MITEEc1, ISSty2, ISSen6, ISEc9, ISKox3	*qnrS1*, *bla_CTX-M_*, *sul2*, *dfrA14*, *tet(A*), *aph(6)-Id*, *aph(3'')-Ib*, *gyrA*, *mdtK*, *sdiA*, *crp*	INS0017009
TPS12	*Salmonella enterica*	Typhi	ST 1	13	9:d:-	IncFIB(K), IncFIB(pHCM2)	MITEEc1, ISSen6, ISEc9, ISKox3, IS5075, ISVsa5, ISKpn19	*qnrS1*, *sul2*, *dfrA14*, *tet(A*), *aph(6)-Id*, *gyrA*, *mdtK*, *sdiA*, *crp*	INS0017010
TPS13	*Salmonella enterica*	Gallinarum	ST 1	13	9:-:-	IncFIB(K), IncFIB(pHCM2)	MITEEc1, ISSty2, ISSen6, ISEc9, ISKox3, IS5075, ISKpn19	*qnrS1*, *bla_CTX-M_*, *sul2*, *dfrA14*, *tet(A*), *aph(6)-Id*, *gyrA*, *mdtK*, *sdiA*, *crp*	INS0017011
TPS14	*Salmonella enterica*	Typhi	ST 1	13	9:d:-	IncFIB(K), IncFIB(pHCM2)	MITEEc1, ISSty2, ISSen6, ISEc9, ISKox3, IS5075, ISKpn19	*qnrS1*, *bla_CTX-M_*, *sul2*, *dfrA14*, *tet(A*), *aph(6)-Id*, *gyrA*, *mdtK*, *sdiA*, *crp*	INS0017012
TPS15	*Salmonella enterica*	Typhi	ST 1	13	9:d:-	IncFIB(K), IncFIB(pHCM2)	MITEEc1, ISSty2, ISSen6, ISEc9, ISKox3, IS5075	*qnrS1*, *bla_CTX-M_*, *sul2*, *dfrA14*, *tet(A*), *aph(6)-Id*, *gyrA*, *mdtK*, *sdiA*, *crp*	INS0017013
TPS16	*Salmonella enterica*	Typhi	ST 1	13	9:d:-	IncFIB(K), IncFIB(pHCM2)	MITEEc1, ISSty2, ISSen6, ISEc9, IS5075	*qnrS1*, *bla_CTX-M_*, *sul2*, *dfrA14*, *tet(A*), *aph(6)-Id*, *gyrA*, *mdtK*, *sdiA*	INS0017084
TPS17	*Salmonella enterica*	Typhi	ST 1	13	9:d:-	IncFIB(K), IncFIB(pHCM2)	MITEEc1, ISSty2, ISSen6, ISEc9, ISKox3, IS5075	*qnrS1*, *bla_CTX-M_*, *sul2*, *dfrA14*, *tet(A*), *aph(6)-Id*, *gyrA*, *mdtK*, *sdiA*, *crp*	INS0017014
TPS18	*Salmonella enterica*	Typhi	ST 1	13	9:d:-	IncFIB(K), IncFIB(pHCM2)	MITEEc1, ISSty2, ISSen6, ISKox3, IS5075, ISKpn19	*qnrS1*, *bla_CTX-M_*, *sul2*, *dfrA14*, *tet(A*), *aph(6)-Id*, *gyrA*, *mdtK*, *sdiA*, *crp*	INS0017015
TPS19	*Salmonella enterica*	Typhi	ST 1	13	4:d:-	IncFIB(K), IncFIB(pHCM2)	MITEEc1, ISSty2, ISSen6, ISEc9, ISKox3, ISVsa5, IS26	*qnrS1*, *bla_CTX-M_*, *tet(A*), *gyrA*, *mdtK*, *sdiA, tufA*	INS0017016
TPS20	*Salmonella enterica*	Typhi	ST 1	13	9:d:-	IncFIB(K), IncFIB(pHCM2)	MITEEc1, ISSty2, ISSen6, ISEc9, ISKox3, IS5075	*qnrS1*, *bla_CTX-M_*, *sul2*, *tet(A*), *aph(6)-Id*, *gyrA*, *mdtK*, *sdiA*, *crp*	INS0017018
TPS21	*Salmonella enterica*	Typhi	ST 1	13	9:d:-	IncFIB(K), IncFIB(pHCM2)	MITEEc1, ISSty2, ISSen6, ISEc9, ISKox3, IS5075, ISVsa5	*qnrS1*, *bla_CTX-M_*, *sul2*, *dfrA14*, *tet(A*), *aph(6)-Id*, *aph(3'')-Ib*, *gyrA*, *mdtK*, *sdiA*, *crp*	INS0017019
TPS22	*Salmonella enterica*	Typhi	ST 1	13	9:d:-	IncFIB(K), IncFIB(pHCM2)	MITEEc1, ISSty2, ISSen6, ISEc9, IS5075	*qnrS1*, *bla_CTX-M_*, *sul2*, *dfrA14*, *tet(A*), *aph(6)-Id*, *gyrA*, *mdtK*, *sdiA*, *crp*	INS0017020
TPS23	*Salmonella enterica*	Typhi	ST 1	13	9:d:-	IncFIB(K), IncFIB(pHCM2)	MITEEc1, ISSty2, ISEc9, IS5075, ISKpn19	*qnrS1*, *bla_CTX-M_*, *sul2*, *dfrA14*, *tet(A*), *aph(6)-Id*, *gyrA*, *mdtK*, *sdiA*, *crp*	INS0017021
TPS24	*Salmonella enterica*	Typhi	ST 1	13	9:d:-	IncFIB(K), IncFIB(pHCM2)	MITEEc1, ISSty2, ISSen6, ISEc9, IS5075, ISKox3	*qnrS1*, *bla_CTX-M_*, *sul2*, *dfrA14*, *tet(A*), *aph(6)-Id*, *gyrA*, *mdtK*, *sdiA*, *crp*	INS0017022
TPS25	*Salmonella enterica*	Typhi	ST 1	13	9:d:-	IncFIB(K), IncFIB(pHCM2)	MITEEc1, ISSty2, ISSen6, ISEc9, IS5075, ISKox3, ISKpn19, IS26	*qnrS1*, *bla_CTX-M_*, *sul2*, *dfrA14*, *tet(A*), *aph(6)-Id*, *gyrA*, *mdtK*, *sdiA*, *crp*	INS0017023
TPS26	*Salmonella enterica*	Typhi	ST 1	13	9:d:-	IncFIB(K), IncFIB(pHCM2)	MITEEc1, ISSty2, ISSen6, ISEc9, IS5075, ISKox3, ISKpn19	*qnrS1*, *bla_CTX-M_*, *sul2*, *dfrA14*, *tet(A*), *aph(6)-Id*, *gyrA*, *mdtK*, *sdiA*, *crp*	INS0017024
TPS27	*Salmonella enterica*	Typhi	ST 1	13	9:d:-	IncFIB(K), IncFIB(pHCM2)	MITEEc1, ISSty2, ISSen6, ISEc9, ISKox3, IS5075, ISVsa5	*qnrS1*, *bla_CTX-M_*, *sul2*, *dfrA14*, *tet(A*), *aph(6)-Id*, *gyrA*, *mdtK*, *sdiA*, *crp*	INS0017025
TPS28	*Salmonella enterica*	Typhi	ST 1	13	9:d:-	IncFIB(K), IncFIB(pHCM2)	MITEEc1, ISSty2, ISSen6, ISEc9, ISKox3, IS5075, ISVsa5	*qnrS1*, *bla_CTX-M_*, *sul2*, *dfrA14*, *tet(A*), *aph(6)-Id*, *gyrA*, *mdtK*, *sdiA*, *crp*	INS0017026
TPS29	*Salmonella enterica*	Typhi	ST 1	13	9:d:-	IncFIB(K), IncFIB(pHCM2)	MITEEc1, ISSty2, ISSen6, ISEc9, IS5075	*qnrS1*, *bla_CTX-M_*, *sul2*, *dfrA14*, *tet(A*), *aph(6)-Id*, *gyrA*, *mdtK*, *sdiA*, *crp*	INS0017027
TPS30	*Salmonella enterica*	Typhi	ST 1	13	9:d:-	IncFIB(K), IncFIB(pHCM2)	MITEEc1, ISSty2, ISSen6, ISEc9, IS5075, ISVsa5	*qnrS1*, *bla_CTX-M_*, *sul2*, *dfrA14*, *tet(A*), *aph(6)-Id*, *gyrA*, *mdtK*, *sdiA*, *crp*	INS0017029
TPS31	*Salmonella enterica*	Typhi	ST 1	13	9:d:-	IncFIB(K), IncFIB(pHCM2)	MITEEc1, ISSty2, ISSen6, ISEc9, IS5075, ISVsa5, ISKox3, ISKpn19	*qnrS1*, *bla_CTX-M_*, *sul2*, *dfrA14*, *tet(A*), *aph(6)-Id*, *gyrA*, *mdtK*, *sdiA*, *crp*	INS0017030
TPS32	*Salmonella enterica*	Typhi	ST 1	13	9:d:-	IncFIB(K), IncFIB(pHCM2)	MITEEc1, ISSty2, ISSen6, ISEc9, IS5075, ISVsa5, ISKox3, ISKpn19	*qnrS1*, *bla_CTX-M_*, *sul2*, *dfrA14*, *tet(A*), *aph(6)-Id*, *gyrA*, *mdtK*, *sdiA*, *crp*	INS0017031
TPS33	*Salmonella enterica*	Typhi	ST 1	13	9:d:-	IncFIB(K), IncFIB(pHCM2)	MITEEc1, ISSty2, ISSen6, ISEc9, IS5075, ISVsa5, ISKpn19	*qnrS1*, *bla_CTX-M_*, *sul2*, *dfrA14*, *tet(A*), *aph(6)-Id*, *gyrA*, *mdtK*, *sdiA*, *crp*	INS0017032
TPS34	*Salmonella enterica*	Typhi	ST 1	13	9:d:-	IncFIB(K), IncFIB(pHCM2)	MITEEc1, ISSty2, IS5075, ISVsa5, ISKox3, ISKpn19	*qnrS1*, *bla_CTX-M_*, *sul2*, *dfrA14*, *tet(A*), *aph(6)-Id*, *gyrA*, *mdtK*, *sdiA*, *crp*	INS0017033
TPS35	*Salmonella enterica*	Typhi	ST 1	13	9:d:-	IncFIB(K), IncFIB(pHCM2)	MITEEc1, ISSty2, ISSen6, IS5075, ISKox3, ISKpn19	*qnrS1*, *bla_CTX-M_*, *sul2*, *dfrA14*, *tet(A*), *aph(6)-Id*, *gyrA*, *mdtK*, *sdiA*	INS0017034
TPS36	*Salmonella enterica*	Typhi	ST 1	13	9:d:-	IncFIB(K), IncFIB(pHCM2)	MITEEc1, ISSty2, ISSen6, ISEc9, IS5075, ISKox3,	*qnrS1*, *bla_CTX-M_*, *dfrA14*, *tet(A*), *aph(6)-Id*, *aph(3'')-Ib*, *gyrA*, *mdtK*, *sdiA*, *crp*	INS0017035
TPS37	*Salmonella enterica*	Typhi	ST 1	13	9:d:-	IncFIB(K), IncFIB(pHCM2)	MITEEc1, ISSty2, ISSen6, ISEc9, IS5075, ISVsa5, ISKox3, ISKpn19	*qnrS1*, *bla_CTX-M_*, *sul2*, *dfrA14*, *tet(A*), *aph(6)-Id*, *gyrA*, *mdtK*, *sdiA*, *crp*	INS0017036
TPS38	*Salmonella enterica*	Typhi	ST 1	13	9:d:-	IncFIB(K), IncFIB(pHCM2)	MITEEc1, ISSty2, ISSen6, ISEc9, IS5075, ISVsa5, ISKox3, ISKpn19	*qnrS1*, *bla_CTX-M_*, *sul2*, *dfrA14*, *tet(A*), *aph(6)-Id*, *gyrA*, *mdtK*, *sdiA*	INS0017037
TPS39	*Salmonella enterica*	Typhi	ST 1	13	9:d:-	IncFIB(K), IncFIB(pHCM2)	MITEEc1, ISSty2, ISSen6, ISEc9, IS5075, ISKox3,	*qnrS1*, *bla_CTX-M_*, *sul2*, *dfrA14*, *tet(A*), *aph(6)-Id*, *gyrA*, *mdtK*, *sdiA*, *crp*	INS0017038
TPS40	*Salmonella enterica*	Typhi	ST 1	13	9:d:-	IncFIB(K), IncFIB(pHCM2)	MITEEc1, ISSty2, ISSen6, ISEc9, IS5075, ISVsa5, ISKox3	*qnrS1*, *bla_CTX-M_*, *sul2*, *dfrA14*, *tet(A*), *aph(6)-Id*, *gyrA*, *mdtK*, *sdiA*, *crp*	INS0017040
TPS41	*Salmonella enterica*	Typhi	ST 1	13	9:d:-	IncFIB(K), IncFIB(pHCM2)	MITEEc1, ISSty2, ISEc9, IS5075, ISKox3,	*qnrS1*, *bla_CTX-M_*, *sul2*, *tet(A*), *aph(6)-Id*, *gyrA*, *mdtK*, *sdiA*, *crp*	INS0017041
TPS42	*Salmonella enterica*	Typhi	ST 1	13	9:d:-	IncFIB(K), IncFIB(pHCM2)	MITEEc1, ISSty2, ISSen6, ISEc9, IS5075, ISKox3	*qnrS1*, *bla_CTX-M_*, *sul2*, *tet(A*), *aph(6)-Id*, *gyrA*, *mdtK*, *sdiA*, *crp*	INS0017042
TPS43	*Salmonella enterica*	Typhi	ST 1	13	9:d:-		MITEEc1, ISSty2, ISSen6	*mdtK, sdiA, crp*	INS0017043
TPS44	*Salmonella enterica*	Typhi	ST 1	13	9:d:-	IncFIB(K), IncFIB(pHCM2)	MITEEc1, ISSty2, ISSen6, ISEc9, IS5075	*qnrS1*, *bla_CTX-M_*, *sul2*, *tet(A*), *aph(6)-Id*, *gyrA*, *mdtK*, *sdiA*, *crp*	INS0017044
TPS45	*Salmonella enterica*	Typhi	ST 1	13	9:d:-	IncFIB(K), IncFIB(pHCM2)	MITEEc1, ISSty2, ISSen6, ISEc9, IS5075, ISKox3, ISKpn19	*qnrS1*, *bla_CTX-M_*, *sul2*, *tet(A*), *aph(6)-Id*, *gyrA*, *mdtK*, *sdiA*, *crp*	INS0017045
TPS46	*Salmonella enterica*	Typhi	ST 1	13	9:d:-	IncFIB(K), IncFIB(pHCM2)	MITEEc1, ISSty2, ISSen6, ISEc9, IS5075, IS26	*qnrS1*, *bla_CTX-M_*, *sul2*, *dfrA14*, *tet(A*), *aph(6)-Id*, *gyrA*, *mdtK*, *sdiA*, *crp*	INS0017046
TPS47	*Salmonella enterica*	Typhi	ST 1	13	9:d:-	IncFIB(K), IncFIB(pHCM2) IncQ1	MITEEc1, ISSty2, ISSen6, IS26	*qnrS1, aph(6)-Id, sul2, mdtK, sdiA, crp*	INS0017047
TPS48	*Salmonella enterica*	Typhi	ST 1	13	9:d:-	IncFIB(K), IncFIB(pHCM2)	MITEEc1, ISSty2, ISSen6, ISEc9, IS5075, ISVsa5, ISKox3, ISKpn19	*qnrS1*, *bla_CTX-M_*, *sul2*, *dfrA14*, *tet(A*), *aph(6)-Id*, *gyrA*, *mdtK*, *sdiA*, *crp*	INS0017048
TPS49	*Salmonella enterica*	Typhi	ST 1	13	9:d:-	IncFIB(K), IncFIB(pHCM2)	MITEEc1, ISSty2, ISSen6, IS5075, ISKox3	*qnrS1*, *sul2*, *tet(A*), *aph(6)-Id*, *gyrA*, *sdiA*, *crp*	INS0017049
TPS50	*Salmonella enterica*	Typhi	ST 1	13	9:d:-	IncFIB(K), IncFIB(pHCM2)	MITEEc1, ISSty2, ISSen6, ISEc9, IS5075, ISVsa5	*qnrS1*, *bla_CTX-M_*, *sul2*, *tet(A*), *aph(6)-Id*, *gyrA*, *mdtK*, *sdiA*, *crp*	INS0017051
TPS51	*Salmonella enterica*	Typhi	ST 1	13	9:d:-	IncFIB(K), IncFIB(pHCM2)	MITEEc1, ISSty2, ISSen6, IS5075, ISKox3, ISKpn19, IS26	*qnrS1, dfrA14, bla_CTX-M_*, *sul2*, *tet(A*), *aph(6)-Id*, *mdtK*, *sdiA*	INS0017052
TPS52	*Salmonella enterica*	Typhi	ST 1	13	9:d:-	IncFIB(K), IncFIB(pHCM2)	MITEEc1, ISSty2, ISSen6, ISEc9, IS5075, ISVsa5, ISKox3	*qnrS1*, *bla_CTX-M_*, *sul2*, *tet(A*), *aph(6)-Id*, *gyrA*, *mdtK*, *sdiA*, *crp*	INS0017053
TPS53	*Salmonella enterica*	Typhi	ST 1	13	9:d:-	IncFIB(K), IncFIB(pHCM2)	MITEEc1, ISSty2, ISSen6, ISEc9, IS5075, ISVsa5, ISKox3, ISKpn19	*qnrS1*, *sul2*, *tet(A*), *aph(6)-Id*, *gyrA*, *mdtK*, *sdiA*, *crp*	INS0017054
TPS54	*Salmonella enterica*	Typhi	ST 1	13	9:d:-	IncFIB(K), IncFIB(pHCM2)	MITEEc1, ISSty2, ISSen6, ISEc9, IS5075, ISVsa5, ISKox3, ISKpn19, IS26	*qnrS1*, *bla_CTX-M_*, *sul2*, *aph(6)-Id*, *gyrA*, *mdtK*, *sdiA*, *crp*	INS0017055
TPS55	*Salmonella enterica*	Typhi	ST 1	13	9:d:-	IncFIB(K), IncFIB(pHCM2)	MITEEc1, ISSty2, ISSen6, ISEc9, IS5075, ISKox3, ISKpn19	*qnrS1*, *bla_CTX-M_*, *sul2*, *aph(6)-Id*, *gyrA*, *mdtK*, *sdiA*, *crp*	INS0017056
TPS56	*Salmonella enterica*	Typhi	ST 1	13	9:d:-	IncFIB(K), IncFIB(pHCM2)	MITEEc1, ISSty2, ISSen6, ISEc9, IS5075, ISVsa5, ISKox3, ISKpn19	*bla_CTX-M_*, *sul2*, *tet(A*), *aph(6)-Id*, *gyrA*, *mdtK*, *sdiA*, *crp*	INS0017057
TPS57	*Salmonella enterica*	Typhi	ST 1	13	9:d:-	IncFIB(K), IncFIB(pHCM2)	MITEEc1, ISSty2, ISSen6, ISEc9, IS5075, ISVsa5, ISKox3, ISKpn19	*qnrS1*, *sul2*, *tet(A*), *aph(6)-Id*, *gyrA*, *mdtK*, *sdiA*, *crp*	INS0017058
TPS58	*Salmonella enterica*	Typhi	ST 1	13	9:d:-	IncFIB(K), IncFIB(pHCM2)	MITEEc1, ISSty2, ISSen6, ISEc9, IS5075, ISVsa5, ISKox3	*qnrS1*, *bla_CTX-M_*, *sul2*, *tet(A*), *aph(6)-Id*, *gyrA*, *mdtK*, *sdiA*, *crp*	INS0017059
TPS59	*Salmonella enterica*	Typhi	ST 1	13	9:d:-	IncFIB(K), IncFIB(pHCM2)	MITEEc1, ISSty2, ISSen6, ISEc9, IS5075, ISKox3	*qnrS1*, *bla_CTX-M_*, *sul2*, *tet(A*), *aph(6)-Id*, *gyrA*, *mdtK*, *sdiA*, *crp*	INS0017060
TPS60	*Salmonella enterica*	Typhi	ST 1	13	9:d:-	IncFIB(K), IncFIB(pHCM2)	MITEEc1, ISSty2, ISSen6, ISEc9, IS5075, ISVsa5, ISKox3	*qnrS1*, *bla_CTX-M_*, *sul2*, *tet(A*), *aph(6)-Id*, *aph(3'')-Ib*, *gyrA*, *mdtK*, *sdiA*, *crp*	INS0017062
TPS61	*Salmonella enterica*	Typhi	ST 1	13	9:d:-	IncFIB(K), IncFIB(pHCM2)	MITEEc1, ISSty2, ISEc9, IS5075, ISVsa5, ISKox3, ISKpn19	*qnrS1*, *bla_CTX-M_*, *sul2*, *tet(A*), *aph(6)-Id*, *gyrA*, *mdtK*, *sdiA*, *crp*	INS0017063
TPS62	*Salmonella enterica*	Typhi	ST 1	13	9:d:-	IncFIB(K), IncFIB(pHCM2)	MITEEc1, ISSty2, ISSen6, ISEc9, IS5075, ISKox3, ISKpn19	*qnrS1*, *bla_CTX-M_*, *sul2*, *aph(6)-Id*, *gyrA*, *mdtK*, *sdiA*, *crp*	INS0017064
TPS63	*Salmonella enterica*	Typhi	ST 1	13	9:d:-	IncFIB(K), IncFIB(pHCM2)	MITEEc1, ISSty2, ISSen6, ISEc9, IS5075, ISKox3, ISKpn19	*qnrS1*, *sul2*, *tet(A*), *aph(6)-Id*, *gyrA*, *mdtK*, *sdiA*, *crp*	INS0017065
TPS64	*Salmonella enterica*	Typhi	ST 1	13	9:d:-	IncFIB(K), IncFIB(pHCM2)	MITEEc1, ISSen6, IS5075, ISKox3, ISKpn19	*qnrS1*, *bla_CTX-M_*, *sul2*, *tet(A*), *aph(6)-Id*, *gyrA*, *mdtK*, *sdiA*, *crp*	INS0017066
TPS65	*Salmonella enterica*	Typhi	ST 1	13	9:d:-	IncFIB(K), IncFIB(pHCM2)	MITEEc1, ISSty2, ISSen6, ISEc9, IS5075, ISVsa5, ISKox3	*qnrS1*, *bla_CTX-M_*, *sul2*, *aph(6)-Id*, *gyrA*, *mdtK*, *sdiA*, *crp*	INS0017067
TPS66	*Salmonella enterica*	Typhi	ST 1	13	9:d:-	IncFIB(K), IncFIB(pHCM2)	MITEEc1, ISSty2, ISSen6, ISEc9, IS5075, ISKox3	*qnrS1*, *bla_CTX-M_*, *sul2*, *tet(A*), *aph(6)-Id*, *gyrA*, *mdtK*, *sdiA*, *crp*	INS0017068
TPS67	*Salmonella enterica*	Typhi	ST 1	13	9:d:-	IncFIB(K), IncFIB(pHCM2)	MITEEc1, ISSty2, ISSen6, ISEc9, ISKox3	*qnrS1*, *bla_CTX-M_*, *sul2*, *tet(A*), *aph(6)-Id*, *aph(3'')-Ib*, *gyrA*, *mdtK*, *sdiA*, *crp*	INS0017069
TPS68	*Salmonella enterica*	Typhi	ST 1	13	9:d:-	IncFIB(K), IncFIB(pHCM2)	MITEEc1, ISSty2, ISSen6, ISEc9, IS5075, ISVsa5, ISKpn19	*qnrS1*, *bla_CTX-M_*, *sul2*, *tet(A*), *aph(6)-Id*, *gyrA*, *mdtK*, *sdiA*, *crp*	INS0017070
TPS69	*Salmonella enterica*	Typhi	ST 1	13	9:d:-	IncFIB(K), IncFIB(pHCM2)	MITEEc1, ISSty2, ISSen6, ISEc9, IS5075, ISVsa5, ISKox3	*qnrS1*, *bla_CTX-M_*, *sul2*, *tet(A*), *aph(6)-Id*, *gyrA*, *mdtK*, *sdiA*, *crp*	INS0017071
TPS70	*Salmonella enterica*	Typhi	ST 1	13	9:d:-	IncFIB(K), IncFIB(pHCM2)	MITEEc1, ISSty2, ISSen6, ISEc9, IS5075, ISVsa5, ISKox3	*qnrS1*, *sul2*, *tet(A*), *aph(6)-Id*, *gyrA*, *mdtK*, *sdiA*, *crp*	INS0017073
TPS71	*Salmonella enterica*	Typhi	ST 1	13	9:d:-	IncFIB(K), IncFIB(pHCM2)	MITEEc1, ISSty2, ISSen6, ISEc9, IS5075, ISVsa5, ISKox3, ISKpn19	*qnrS1*, *bla_CTX-M_*, *sul2*, *tet(A*), *aph(6)-Id*, *gyrA*, *mdtK*, *sdiA*, *crp*	INS0017074
TPS72	*Salmonella enterica*	Typhi	ST 1	13	9:d:-	IncFIB(K), IncFIB(pHCM2)	MITEEc1, ISSty2, ISSen6, ISEc9, IS5075, ISKox3, ISKpn19	*qnrS1*, *sul2*, *tet(A*), *aph(6)-Id*, *gyrA*, *sdiA*, *crp*	INS0017075
TPS73	*Salmonella enterica*	Typhi	ST 1	13	9:d:-	IncFIB(K), IncFIB(pHCM2)	MITEEc1, ISSty2, ISEc9, IS5075, ISVsa5, ISKox3	*qnrS1*, *bla_CTX-M_*, *sul2*, *tet(A*), *aph(6)-Id*, *gyrA*, *mdtK*, *sdiA*, *crp*	INS0017076
TPS74	*Salmonella enterica*	Typhi	ST 1	13	9:d:-	IncFIB(K), IncFIB(pHCM2)	MITEEc1, ISSty2, ISSen6, ISEc9, IS5075, ISKox3	*qnrS1*, *bla_CTX-M_*, *sul2*, *tet(A*), *aph(6)-Id*, *gyrA*, *mdtK*, *sdiA*, *crp*	INS0017077
TPS75	*Salmonella enterica*	Typhi	ST 1	13	9:d:-	IncFIB(K), IncFIB(pHCM2)	MITEEc1, ISSty2, ISSen6, ISEc9, IS5075, ISVsa5, ISKox32	*qnrS1*, *bla_CTX-M_*, *sul2*, *tet(A*), *aph(6)-Id*, *gyrA*, *mdtK*, *sdiA*, *crp*	INS0017078
Unipath14	*Salmonella enterica*	Typhi	ST1	13	9:d:-	IncFIB(K), IncFIB(pHCM2)	ISEc9, ISSen6, ISVsa5, ISKox3, IS5075, ISSty2, MITEEc1	*qnrS1*, *bla_CTX-M_*, *sul2*, *dfrA14*, *tet(A*), *aph(6)-Id*, *aph(3'')-Ib*, *gyrA*, *mdtK*, *sdiA*, *crp*	INS0017082
Unipath22	*Salmonella enterica*	Typhi	ST1	13	9:d:-	IncFIB(K), IncFIB(pHCM2)	ISEc9, ISSen6, ISVsa5, ISKox3, IS5075, ISSty2, MITEEc1	*qnrS1*, *bla_CTX-M_*, *sul2*, *dfrA14*, *tet(A*), *aph(6)-Id*, *aph(3'')-Ib*, *gyrA*, *mdtK*, *sdiA*, *crp*	INS0017086
Unipath48	*Salmonella enterica*	Typhi	ST1	13	9:d:-	-	ISSen6, ISSty2, MITEEc1	*mdtK, sdiA*, *crp*	INS0017094
Unipath55	*Salmonella enterica*	Typhi	ST1	13	9:d:-	IncFIB(K), IncFIB(pHCM2)	ISSty2, ISEc9, ISKpn19, ISSen6, ISVsa5, ISKox3, IS5075, MITEEc1	*qnrS1*, *bla_CTX-M_*, *sul2*, *dfrA14*, *tet(A*), *aph(6)-Id*, *aph(3'')-Ib*, *gyrA*, *mdtK*, *sdiA*, *crp*	INS0017095
Unipath93	*Salmonella enterica*	Typhi	ST1	13	9:d:-	IncFIB(K), IncFIB(pHCM2)	ISEc9, ISKpn19, ISSen6, ISKox3, IS5075, ISSty2, MITEEc1	*qnrS1*, *bla_CTX-M_*, *sul2*, *dfrA14*, *tet(A*), *aph(6)-Id*, *aph(3'')-Ib*, *gyrA*, *mdtK*, *sdiA*, *crp*	INS0017109
Unipath127	*Salmonella enterica*	Typhi	ST1	13	9:d:-	IncFIB(K), IncFIB(pHCM2)	ISEc9, ISKpn19, ISSen6, ISVsa5, ISKox3, IS5075, ISSty2, MITEEc1	*qnrS1*, *bla_CTX-M_*, *sul2*, *dfrA14*, *tet(A*), *aph(6)-Id*, *aph(3'')-Ib*, *gyrA*, *mdtK*, *sdiA*, *crp*	INS0017081
Unipath153	*Salmonella enterica*	Typhi	ST1	13	9:d:-	IncFIB(K), IncFIB(pHCM2)	ISEc9, ISSen6, ISVsa5, ISKox3, IS5075, ISSty2, MITEEc1	*qnrS1*, *bla_CTX-M_*, *sul2*, *dfrA14*, *tet(A*), *aph(6)-Id*, *aph(3'')-Ib*, *gyrA*, *mdtK*, *sdiA*, *crp*	INS0017083
Unipath201	*Salmonella enterica*	Typhi	ST1	13	9:d:-	IncFIB(K), IncFIB(pHCM2)	ISEc9, ISKpn19, ISSen6, ISVsa5, ISKox3, IS5075, ISSty2, MITEEc1	*qnrS1*, *bla_CTX-M_*, *sul2*, *dfrA14*, *tet(A*), *aph(6)-Id*, *aph(3'')-Ib*, *gyrA*, *mdtK*, *sdiA*, *crp*	INS0017085
Unipath250	*Salmonella enterica*	Typhi	ST1	13	9:d:-	IncFIB(K), IncFIB(pHCM2)	ISEc9, ISSen6, ISVsa5, ISKox3, IS5075, ISSty2, MITEEc1	*qnrS1*, *bla_CTX-M_*, *sul2*, *dfrA14*, *tet(A*), *aph(6)-Id*, *aph(3'')-Ib*, *gyrA*, *mdtK*, *sdiA*, *crp*	INS0017087
Unipath255	*Salmonella enterica*	Typhi	ST1	13	9:d:-	IncFIB(K), IncFIB(pHCM2)	ISEc9, ISKpn19, ISSen6, ISVsa5, ISKox3, IS5075, ISSty2, MITEEc1	*qnrS1*, *bla_CTX-M_*, *sul2*, *dfrA14*, *tet(A*), *aph(6)-Id*, *aph(3'')-Ib*, *gyrA*, *mdtK*, *sdiA*, *crp*	INS0017088
Unipath286	*Salmonella enterica*	Typhi	ST1	13	9:d:-	IncFIB(K), IncFIB(pHCM2)	ISEc9, ISKpn19, ISSen6, ISVsa5, ISKox3, IS5075, ISSty2, MITEEc1	*mdtK, sdiA*, *crp*	INS0017089
Unipath292	*Salmonella enterica*	Typhi	ST1	13	9:d:-	IncFIB(K), IncFIB(pHCM2)	ISEc9, ISKpn19, ISSen6, ISVsa5, ISKox3, IS5075, ISSty2, MITEEc1	*qnrS1*, *bla_CTX-M_*, *sul2*, *dfrA14*, *tet(A*), *aph(6)-Id*, *aph(3'')-Ib*, *gyrA*, *mdtK*, *sdiA*, *crp*	INS0017090
Unipath362	*Salmonella enterica*	Typhi	ST1	13	9:d:-	IncFIB(K), IncFIB(pHCM2)	ISEc9, ISSen6, ISVsa5, ISKox3, IS5075, ISSty2, MITEEc1	*qnrS1*, *bla_CTX-M_*, *sul2*, *dfrA14*, *tet(A*), *aph(6)-Id*, *aph(3'')-Ib*, *gyrA*, *mdtK*, *sdiA*, *crp*	INS0017091
Unipath441	*Salmonella enterica*	Typhi	ST1	13	9:d:-	IncFIB(K), IncFIB(pHCM2)	ISEc9, ISKpn19, ISSen6, ISVsa5, ISKox3, IS5075, ISSty2, MITEEc1	*qnrS1*, *bla_CTX-M_*, *sul2*, *dfrA14*, *tet(A*), *aph(6)-Id*, *aph(3'')-Ib*, *gyrA*, *mdtK*, *sdiA*, *crp*	INS0017092
Unipath461	*Salmonella enterica*	Typhi	ST1	13	9:d:-	IncFIB(K), IncFIB(pHCM2)	ISEc9, ISKpn19, ISSen6, ISVsa5, ISKox3, IS5075, ISSty2, MITEEc1	*qnrS1*, *bla_CTX-M_*, *sul2*, *dfrA14*, *tet(A*), *aph(6)-Id*, *aph(3'')-Ib*, *gyrA*, *mdtK*, *sdiA*, *crp*	INS0017093
Unipath535	*Salmonella enterica*	Typhi	ST1	13	9:d:-	ColM3 IncFIB(K), IncFIB(pHCM2)	ISEc9, ISKpn19, ISSen6, ISVsa5, ISKox3, IS5075, ISSty2, MITEEc1	*qnrS1*, *bla_CTX-M_*, *sul2*, *dfrA14*, *tet(A*), *aph(6)-Id*, *aph(3'')-Ib*, *gyrA*, *mdtK*, *sdiA*, *crp*	INS0017096
Unipath549	*Salmonella enterica*	Typhi	ST1	13	9:d:-	IncFIB(K), IncFIB(pHCM2)	ISEc9, ISKpn19, ISSen6, ISVsa5, ISKox3, IS5075, ISSty2, MITEEc1	*qnrS1*, *bla_CTX-M_*, *sul2*, *dfrA14*, *tet(A*), *aph(6)-Id*, *aph(3'')-Ib*, *gyrA*, *mdtK*, *sdiA*, *crp*	INS0017097
Unipath598	*Salmonella enterica*	Typhi	ST1	13	9:d:-	IncFIB(K), IncFIB(pHCM2)	ISEc9, ISKpn19, ISSen6, ISVsa5, ISKox3, IS5075, ISSty2, MITEEc1	*qnrS1*, *bla_CTX-M_*, *sul2*, *dfrA14*, *tet(A*), *aph(6)-Id*, *aph(3'')-Ib*, *gyrA*, *mdtK*, *sdiA*, *crp*	INS0017098
Unipath616	*Salmonella enterica*	Typhi	ST1	13	9:d:-	IncFIB(K), IncFIB(pHCM2)	ISEc9, ISKpn19, ISSen6, ISVsa5, ISKox3, IS5075, ISSty2, MITEEc1	*qnrS1*, *bla_CTX-M_*, *sul2*, *dfrA14*, *tet(A*), *aph(6)-Id*, *aph(3'')-Ib*, *gyrA*, *mdtK*, *sdiA*, *crp*	INS0017099
Unipath670	*Salmonella enterica*	Typhi	ST1	13	9:d:-	IncFIB(K), IncFIB(pHCM2)	ISEc9, ISSen6, ISVsa5, ISKox3, IS5075, ISSty2, MITEEc1	*qnrS1*, *bla_CTX-M_*, *sul2*, *dfrA14*, *tet(A*), *aph(6)-Id*, *aph(3'')-Ib*, *gyrA*, *mdtK*, *sdiA*, *crp*	INS0017101
Unipath690	*Salmonella enterica*	Typhi	ST1	13	9:d:-	IncFIB(K), IncFIB(pHCM2)	ISEc9, ISSen6, ISVsa5, IS5075, ISSty2, MITEEc1	*qnrS1*, *bla_CTX-M_*, *sul2*, *dfrA14*, *tet(A*), *aph(6)-Id*, *aph(3'')-Ib*, *gyrA*, *mdtK*, *sdiA*, *crp*	INS0017102
Unipath696	*Salmonella enterica*	Typhi	ST1	13	9:d:-	IncFIB(K), IncFIB(pHCM2)	ISEc9, ISKpn19, ISSen6, ISVsa5, ISKox3, IS5075, ISSty2, MITEEc1	*qnrS1*, *bla_CTX-M_*, *sul2*, *dfrA14*, *tet(A*), *aph(6)-Id*, *aph(3'')-Ib*, *gyrA*, *mdtK*, *sdiA*, *crp*	INS0017100
Unipath709	*Salmonella enterica*	Typhi	ST1	13	9:d:-	IncFIB(K), IncFIB(pHCM2)	ISEc9, ISKpn19, ISSen6, ISVsa5, ISKox3, IS5075, ISSty2, MITEEc1	*qnrS1*, *bla_CTX-M_*, *sul2*, *dfrA14*, *tet(A*), *aph(6)-Id*, *aph(3'')-Ib*, *gyrA*, *mdtK*, *sdiA*, *crp*	INS0017103
Unipath729	*Salmonella enterica*	Typhi	ST1	13	9:d:-	IncFIB(K), IncFIB(pHCM2)	ISEc9, ISSen6, ISVsa5, IS5075, ISSty2, MITEEc1	*qnrS1*, *bla_CTX-M_*, *sul2*, *dfrA14*, *tet(A*), *aph(6)-Id*, *aph(3'')-Ib*, *gyrA*, *mdtK*, *sdiA*, *crp*	INS0017104
Unipath736	*Salmonella enterica*	Typhi	ST1	13	9:d:-	IncFIB(K), IncFIB(pHCM2)	ISEc9, ISKpn19, ISSen6, ISVsa5, ISKox3, IS5075, ISSty2, MITEEc1	*qnrS1*, *bla_CTX-M_*, *sul2*, *dfrA14*, *tet(A*), *aph(6)-Id*, *aph(3'')-Ib*, *gyrA*, *mdtK*, *sdiA*, *crp*	INS0017105
Unipath738	*Salmonella enterica*	Typhi	ST1	13	9:d:-	IncFIB(K), IncFIB(pHCM2)	ISEc9, ISKpn19, ISSen6, ISVsa5, ISKox3, IS5075, ISSty2, MITEEc1	*qnrS1*, *bla_CTX-M_*, *sul2*, *dfrA14*, *tet(A*), *aph(6)-Id*, *aph(3'')-Ib*, *gyrA*, *mdtK*, *sdiA*, *crp*	INS0017106
Unipath750	*Salmonella enterica*	Typhi	ST1	13	9:d:-	IncFIB(K), IncFIB(pHCM2)	ISEc9, ISKpn19, ISSen6, ISVsa5, ISKox3, IS5075, ISSty2, MITEEc1	*qnrS1*, *bla_CTX-M_*, *sul2*, *dfrA14*, *tet(A*), *aph(6)-Id*, *aph(3'')-Ib*, *gyrA*, *mdtK*, *sdiA*, *crp*	INS0017107
Unipath828	*Salmonella enterica*	Typhi	ST1	13	9:d:-	IncFIB(K), IncFIB(pHCM2)	ISEc9, ISKpn19, ISSen6, ISVsa5, ISKox3, IS5075, ISSty2, MITEEc1	*qnrS1*, *bla_CTX-M_*, *sul2*, *dfrA14*, *tet(A*), *aph(6)-Id*, *aph(3'')-Ib*, *gyrA*, *mdtK*, *sdiA*, *crp*	INS0017108
Z1	*Salmonella enterica*	Typhi	ST1	13	9:d:-	IncFIB(K), IncFIB(pHCM2)	ISEc9, ISKpn19, ISSen6, ISVsa5, ISKox3, IS5075, ISSty2, MITEEc1	*qnrS1*, *bla_CTX-M_*, *sul2*, *dfrA14*, *tet(A*), *aph(6)-Id*, *aph(3'')-Ib*, *gyrA*, *mdtK*, *sdiA*, *crp*	INS0017110
Z2	*Salmonella enterica*	Typhi	ST1	13	9:d:-	IncFIB(K), IncFIB(pHCM2)	ISEc9, ISKpn19, ISSen6, ISVsa5, ISKox3, IS5075, ISSty2, MITEEc1	*qnrS1*, *bla_CTX-M_*, *sul2*, *dfrA14*, *tet(A*), *aph(6)-Id*, *aph(3'')-Ib*, *gyrA*, *mdtK*, *sdiA*, *crp*	INS0017111
Z3	*Salmonella enterica*	Typhi	ST1	13	9:d:-	IncFIB(K), IncFIB(pHCM2)	ISEc9, ISSen6, ISVsa5, IS5075, ISSty2, MITEEc1	*qnrS1*, *bla_CTX-M_*, *sul2*, *dfrA14*, *tet(A*), *aph(6)-Id*, *aph(3'')-Ib*, *gyrA*, *mdtK*, *sdiA*, *crp*	INS0017112
Z4	*Salmonella enterica*	Typhi	ST1	13	9:d:-	IncFIB(K), IncFIB(pHCM2)	ISEc9, ISKpn19, ISSen6, ISVsa5, ISKox3, IS5075, ISSty2, MITEEc1	*qnrS1*, *bla_CTX-M_*, *sul2*, *dfrA14*, *tet(A*), *aph(6)-Id*, *aph(3'')-Ib*, *gyrA*, *mdtK*, *sdiA*, *crp*	INS0017113
Z5	*Salmonella enterica*	Typhi	ST1	13	9:d:-	IncFIB(K), IncFIB(pHCM2)	ISEc9, ISKpn19, ISSen6, ISVsa5, ISKox3, IS5075, ISSty2, MITEEc1	*qnrS1*, *bla_CTX-M_*, *sul2*, *dfrA14*, *tet(A*), *aph(6)-Id*, *aph(3'')-Ib*, *gyrA*, *mdtK*, *sdiA*, *crp*	INS0017114
Z6	*Salmonella enterica*	Typhi	ST1	13	9:d:-	IncFIB(K), IncFIB(pHCM2)	ISEc9, ISKpn19, ISSen6, ISVsa5, ISKox3, IS5075, ISSty2, MITEEc1	*qnrS1*, *bla_CTX-M_*, *sul2*, *dfrA14*, *tet(A*), *aph(6)-Id*, *aph(3'')-Ib*, *gyrA*, *mdtK*, *sdiA*, *crp*	INS0017115
Z7	*Salmonella enterica*	Typhi	ST1	13	9:d:-	IncFIB(K), IncFIB(pHCM2)	ISEc9, ISKpn19, ISSen6, ISVsa5, ISKox3, IS5075, ISSty2, MITEEc1	*qnrS1*, *bla_CTX-M_*, *sul2*, *dfrA14*, *tet(A*), *aph(6)-Id*, *aph(3'')-Ib*, *gyrA*, *mdtK*, *sdiA*, *crp*	INS0017116
Z8	*Salmonella enterica*	Typhi	ST1	13	9:d:-	IncFIB(K), IncFIB(pHCM2)	ISEc9, ISPre3, ISSen6, ISVsa5, ISKox3, IS5075, ISSty2, MITEEc1	*qnrS1*, *bla_CTX-M_*, *sul2*, *tet(A*), *aph(6)-Id*, *gyrA*, *mdtK*, *sdiA*, *crp,rsmA*	INS0017117
Z9	*Salmonella enterica*	Typhi	ST1	13	9:d:-	IncFIB(K), IncFIB(pHCM2)	ISEc9, ISKpn19, ISSen6, ISVsa5, ISKox3, IS5075, ISSty2, MITEEc1	*qnrS1*, *bla_CTX-M_*, *sul2*, *dfrA14*, *tet(A*), *aph(6)-Id*, *aph(3'')-Ib*, *gyrA*, *mdtK*, *sdiA*, *crp*	INS0017118
Z10	*Salmonella enterica*	Typhi	ST1	13	9:d:-	IncFIB(K), IncFIB(pHCM2)	ISEc9, ISSen6, ISVsa5, IS5075, ISSty2, MITEEc1	*qnrS1*, *bla_CTX-M_*, *sul2*, *dfrA14*, *tet(A*), *aph(6)-Id*, *aph(3'')-Ib*, *gyrA*, *mdtK*, *sdiA*, *crp*	INS0017119

^*a*^ “-”, not available.

### Determining ARGs using WGS

**Fig 3:**
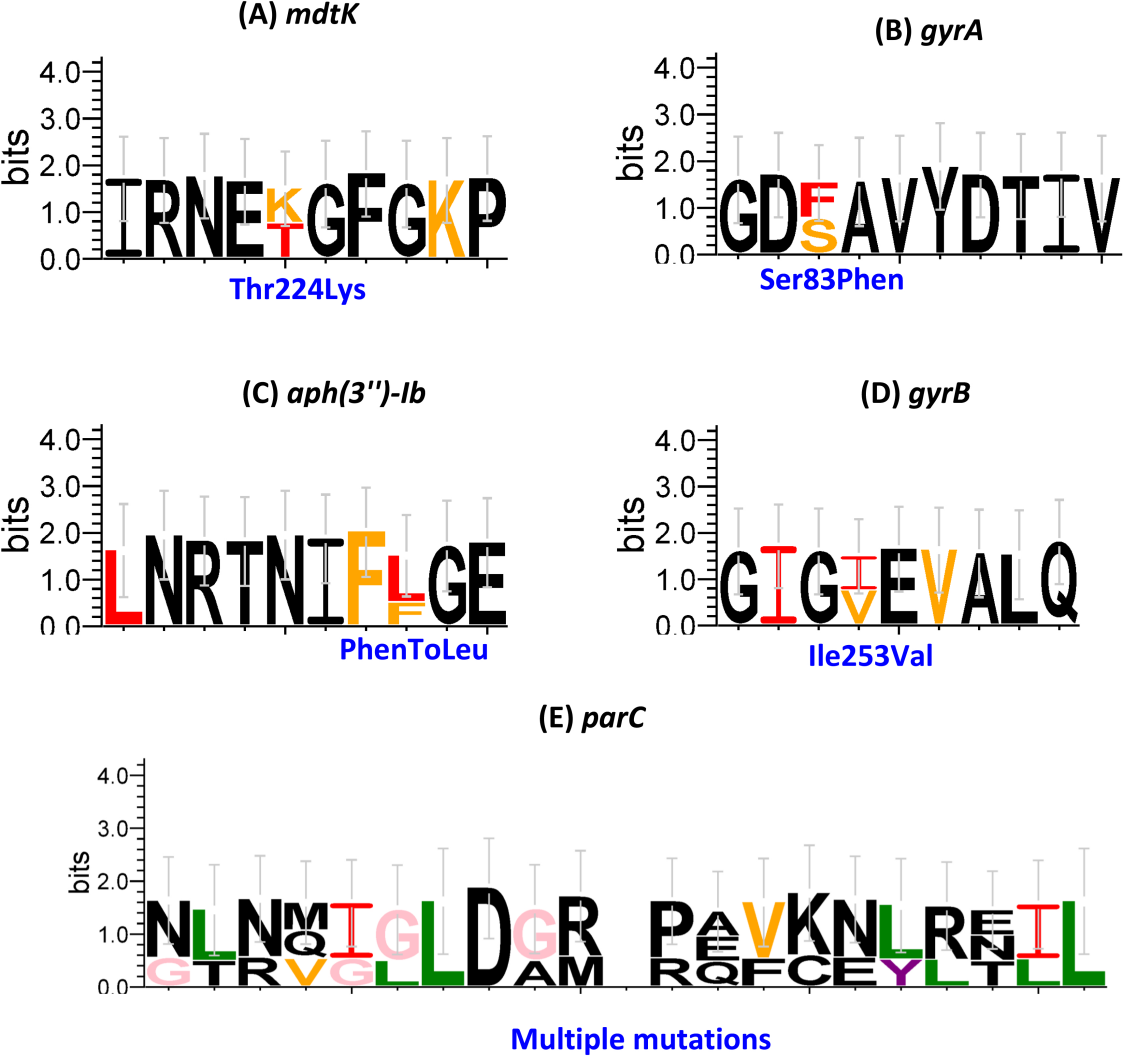
A gallery of sequence logos context for point mutations in *S*. Typhi isolates. (**A**) *mdtk*: Thr224Lys point mutation, (**B**) *gyrA*: Ser83Phen point mutation, (**C**) *aph(3'')-Ib*: Phenylalanine (**F**) to Leucine (**L**) point mutation, (**D**) *gyrB*: Ile253Val point mutation, and (**E**) *parC*: multiple mutations.

A total of 14 ARGs were found to be either on plasmids or chromosomes, and they confer resistance to 11 different categories of antibiotics: aminoglycoside, *β*-lactam antibiotics (aminopenicillin, cephalosporin, carbapenam, and penicillins), macrolides, phenicol, polymyxin, FQs, sulfonamides-trimethoprim, tetracycline, other (fosfomycin), and others. The 122 isolates have genomes that contain 1–11 genes each. The details are described in detail based on the various antimicrobial classes. Aminoglycoside resistance genes: *aph(3'')-Ib* and *aph(6)-Id* were found in 49/122 (40.16%) and 116/122 (95.08%) isolates, respectively. One *β*-lactam resistance gene was identified as *bla*_CTX-M_ and was found only in 109/122 (89.39%) (Table 1). The quinolone resistance determining region (QRDR), which contains chromosomal mutations in *gyrA* and *parC*, is primarily attributed to FQ resistance. A total of 115 *S*. Typhi isolates exhibited notable *gyrA* mutations at the 83-codon level, with amino acid modifications off Ser83Phen in 112/122 (91.80%), Ser83Leu in 2/122 (1.64%), and Ser83Tyr in 1/122 (0.82%), respectively (Fig. 3). *gyrB* mutations at the 252-codon level with amino acid modifications from Ile253Val in 122/122 (100%). In *parC*, 8/122 (6.56%) isolates exhibited multiple mutations (Fig. 3). Plasmid-mediated quinolone resistance (PMQR), known as PMQR mutation in the *qnrS1* gene, was also observed in 115/122 cases (94.26%). One trimethoprim resistance gene (*dfrA14*) was found in 84/122 isolates (68.85%), whereas one sulfonamide resistance gene (*sul2*) was found in 116/122 isolates (95.08%). The global regulator that represses the MdtEF efflux pump was identified as *crp* in 84/122 (68.85%) isolates. Another efflux pump gene capable of the efflux of FQs and *β*-lactam antibiotics, the *mdtk* efflux gene, with this mutation, was found in 120/122 (98.36%) isolates, with amino acid changes from Thr224Lys (Fig. 3), whereas IncFIB(K) and IncFIB(pHCM2) showed 96.71% in *S*. Typhi isolates (Fig. 2).

### Analysis of concordance and discordance

The genotypic and phenotypic tests indicated an average concordance of 91.69%. The highest (99.18%) concordance between genotypes and phenotypes was observed for doxycycline, pefloxacin, and tigecycline. The strong concordance was observed for amoxycillin/clavulanic acid (98.36%), colistin (98.36%), doxycycline (98.36%), tetracycline (98.36%), cefoperazone-sulbactam (96.72%), cefazolin (96.72%), ampicillin (95.90%), ceftriaxone (95.90%), cotrimoxazole (95.90%), cefixime (95.90%), cefepime (95.08%), and cefotaxime (95.08%). Lower than 95% concordance was observed for amikacin (93.44%), gentamicin (91.80%), ampicillin/sulbactam (93.44%), cefuroxime (91.80%), cefuroxime-axetil (92.62%), ertapenem (92.62%), imipenem (79.51%), meropenem (79.51%), piperacillin/tazobactam (81.15%), azithromycin (74.59%), chloramphenicol (90.98%), ciprofloxacin (94.26%), and levofloxacin (54.92%). However, our study revealed a discordance rate of 8.31%. Levofloxacin (45.08%) and azithromycin (25.41%) showed a higher level of discordance. Ampicillin/sulbactam, cefuroxime, and cefuroxime-axetil exhibited 100% sensitive, whereas cefazolin, ceftriaxone, ampicillin, amikacin, and cefepime exhibited 95%–97%. However, only levofloxacin had a lower sensitivity (54.91%). Notably, 45.08% of the isolates phenotypically susceptible to levofloxacin harbored one mutation or acquired genes associated with resistance to levofloxacin. The positive predictive value (PPV) was found in our data set: 100% for ampicillin, ciprofloxacin, levofloxacin, pefloxacin, and tetracycline. No PPV could be calculated for phenicol, macrolides, and polymyxin and other *β*-lactam antibiotics due to our data set’s lack of phenotypic-resistant isolates. Meanwhile, 86% of *β*-lactams and 66.67% of the tetracycline were found to have the least negative predictive values (NPV). No NPVs were calculated for aminoglycosides and FQs due to the lack of phenotypic-resistant isolates in our data set. Table 2 provides a detailed summary of the data.

**TABLE 2:** Phenotypic and genotypic resistance association among all the 122 isolates of *S*. Typhi^*e*^

Antibiotics class	Antibiotic agents	No. of test results				Concordance (%)	Class-wise	Discordance (%)	Class-wise	[a/(a + c)]*100	Class-wise	[d/(b + d)]*100	Class-wise	[a/(a + b)]*100	Class-wise	[d/(c + d)]*100	Class-wise
		Phenotype: resistant		Phenotype: susceptible						Sensitivity		Specificity		PPV		NPV	
		^*a*^	^*b*^	^*c*^	^*d*^												
Aminoglycoside	AMIKACIN	114	3	5	0	93.44	92.62	6.56	7.38	95.80	94.96	0.00	0.00	97.44	97.41	0.00	0.00
	GENTAMICIN	112	3	7	0	91.80		8.20		94.12		0.00		97.39		0.00	
Beta-Lactams	AMOXY/CLAV	0	2	0	120	98.36	92.02	1.64	7.98	0.00	19.28	98.36	48.62	0.00	19.83	100.00	86.00
	AMPICILLIN	114	1	4	3	95.90		4.10		94.26		0.00		100.00		0.00	
	AMPICILLIN/ SULBACTUM	109	8	0	5	93.44		6.56		0.00		4.10		0.00		100.00	
	CEFUROXIME	109	10	0	3	91.80		8.20		0.00		2.46		0.00		100.00	
	CEFUROXIME-AXETIL	109	9	0	4	92.62		7.38		0.00		3.28		0.00		100.00	
	CEFTRIAXONE	115	2	3	2	95.90		4.10		97.46		50.00		98.29		40.00	
	CEFO-SULBACTAM	0	4	0	118	96.72		3.28		0.00		96.72		0.00		100.00	
	CEFEPIME	113	1	5	3	95.08		4.92		0.00		6.56		0.00		100.00	
	CEFAZOLIN	115	1	3	3	96.72		3.28		97.46		75.00		99.14		50.00	
	CEFIXIME	109	6	0	7	95.08		4.92		0.00		5.74		0.00		100.00	
	CEFOTAXIME	114	1	4	3	95.90		4.10		0.00		5.74		0.00		100.00	
	ERTAPENEM	0	9	0	113	92.62		7.38		0.00		92.62		0.00		100.00	
	IMIPENEM	0	25	0	97	79.51		20.49		0.00		79.51		0.00		100.00	
	MEROPENEM	0	25	0	97	79.51		20.49		0.00		79.51		0.00		100.00	
	PIPERACILLIN-TAZOBACTUM	0	23	0	99	81.15		18.85		0.00		81.15		0.00		100.00	
Macrolides	AZITHROMYCIN	0	31	0	91	74.59	74.59	25.41	25.41	0.00		74.59	74.59	0.00		100.00	
Phenicol	CHLORAMPHENICOL	0	11	0	111	90.98	90.98	9.02	9.02	0.00		90.98	90.98	0.00		100.00	
Polymyxin	COLISTIN	0	2	0	120	98.36	98.36	1.64	1.64	0.00		98.36	98.36	0.00		100.00	
Quinolone	CIPROFLOXACIN	115	0	7	0	94.26	82.79	5.74	17.21	94.26	82.79	0.00		100.00	100.00	0.00	0.00
	LEVOFLOXACIN	67	0	55	0	54.92		45.08		54.92		0.00		100.00		0.00	
	PEFLOXACIN	121	0	1	0	99.18		0.82		99.18		0.00		100.00		0.00	
Sulfonamides	COTRIMOXAZOLE	116	1	4	1	95.90		4.10		96.67		50.00		99.15		20.00	
Tetracycline	TETRACYCINE	119	0	3	0	97.54	98.09	2.46	1.91	97.54	98.77		50.83	100.00	33.61	0.00	66.67
	DOXYCYCINE	119	0	3	0	97.54		2.46		100.00		2.48		0.84		100.00	
	TIGECYCLINE	0	1	0	121	99.18		0.82				99.18		0.00		100.00	
Others	FOSFOMYCIN [FO]	0	1	0	121	99.18		0.82				99.18		0.00		100.00	
**Total**						**91.69**		**8.31**		**92.88**		**51.98**		**35.44**		**68.21**	**91.69**

^*a*^ Genotype resistant.

^*b*^ Genotype susceptible.

^*c*^ Genotype resistant.

^*d*^ Genotype susceptible.

^*e*^ PPV: Positive predicting value, NPV: Negative predicting value.

### Phenotypic resistance to ceftriaxone-resistant *S*. Typhi determined via MBD: *β*-lactam/*β*-lactamase inhibitors (BL/BLI) combination

**Fig 4:**
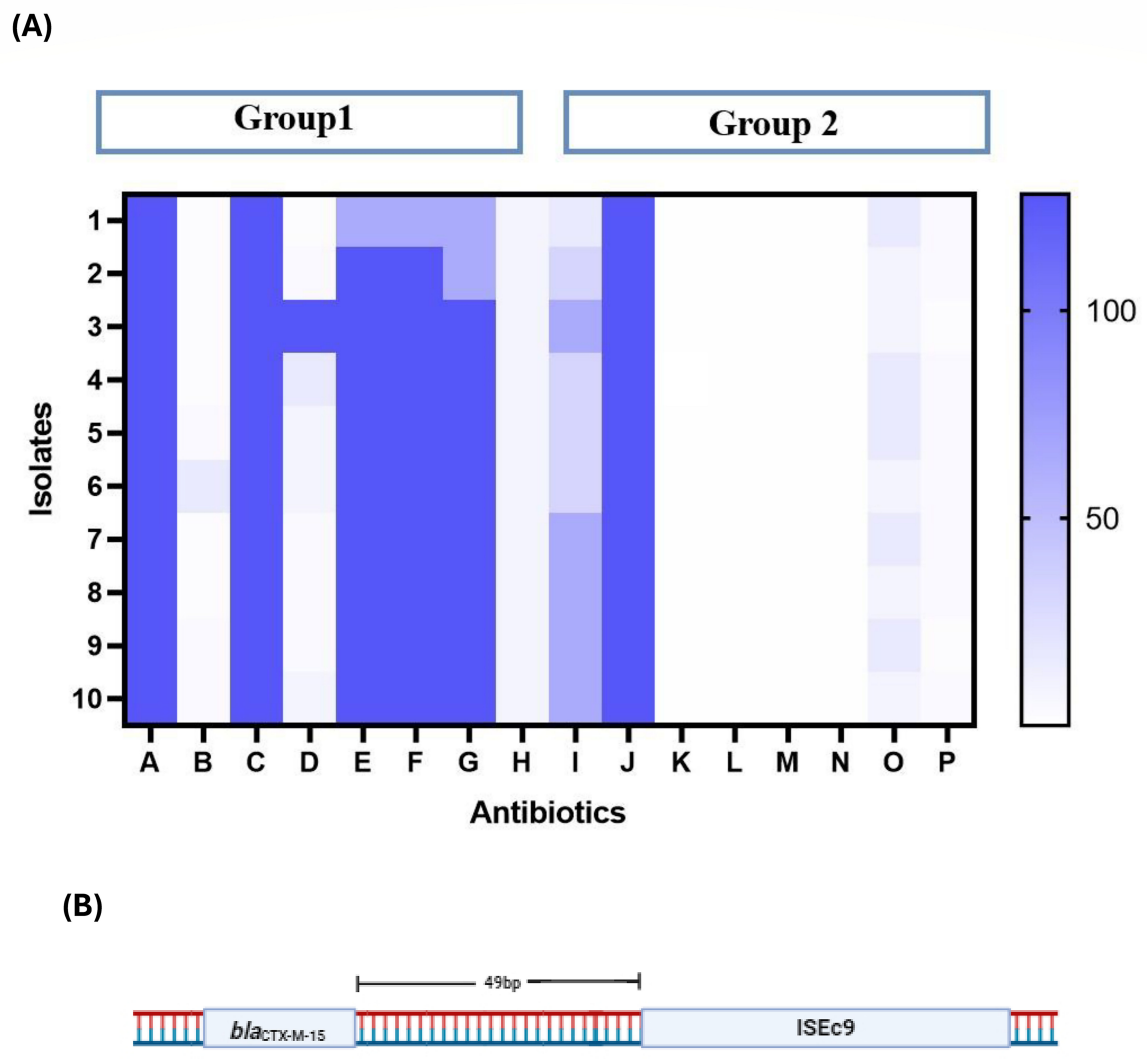
(**A**)The antibiotic susceptibility profile of 10 highly resistant *S*. Typhi isolates to major antibiotics and BL/BLI combinations. (**B**) ISEc9 insertion sequence 49 bp upstream of *bla*_CTX-M-15_.

Ten isolates (TPS01, TPS02, TPS03, TPS04, TPS05, TPS06, TPS07, TPS08, TPS09, and TPS10) that showed the highest resistance toward the major classes of antibiotics were further screened for initial screening for ampicillin, ciprofloxacin, co-trimoxazole, chloramphenicol, ceftriaxone, cefixime, imipenem, and pefloxacin. As shown in Fig. 4, all 10 isolates were resistant to ampicillin (>128 µg/mL), ciprofloxacin (2 µg/mL), co-trimoxazole (>128 µg/mL), ceftazidime (>128 µg/mL), ceftriaxone (>128 µg/mL), cefixime (>128 µg/mL), and imipenem (>128 µg/mL). In comparison, eight isolates were susceptible to chloramphenicol (8 µg/mL). Notably, chloramphenicol showed variation resistance patterns in TPS03 and TPS04 (>128 µg/mL and 16 µg/mL). Although the other eight isolates have shown susceptibility. Similarly, these ceftriaxone-resistant isolates were screened for rare BL/BLI combinations used to treat typhoidal fever. Ceftazidime/avibactam (<0.12 µg/mL), ceftazidime/tazobactam (<0.5 µg/mL), cefexime/tazobactam (<0.5 µg/mL), and ceftriaxone/tazobactam (<0.5 µg/mL) were the combinations that exhibited an increased susceptibility. However, ampicillin/sulbactam (<64 µg/mL) and ceftriaxone/sulbactam (<16 µg/mL) were the combinations that did not exhibit an increase in susceptibility (Fig. 4A).

### Plasmid replicon type and IS element identification

Of the 122 isolates, 109 harbored the *bla*_CTX-M-15_ gene, and one harbored the *bla*_CTX-M-194_ gene. *bla*_CTX-M_ gene was absent in 12 isolates. Of the 122 isolates, 115 harbored IncFIB(K) and IncFIB(pHCM2) plasmid replicon types. Individual isolates included an extra IncQ1 and ColM3 plasmid replicon type. One isolate contained the IncFIB (pHCM2) and IncQ1 plasmid replicon types. One isolate had a single IncFIB(K) plasmid replicon type. Plasmids were shown to be lacking in three isolates. As shown in Fig. 2, the mobile genetic elements MITEEc1, ISSty2, ISSen6, ISEc9, ISKox3, and IS5075 were present in almost all isolates. Interestingly, ISEc9 was shown to be linked with *bla*_CTX-M-15_ in 99 isolates. As demonstrated in Fig. 4B, *bla*_CTX-M-15_ was detected 49 bp upstream of the insertion region (ISEc9), supporting the idea that the insertion sequence (ISEc9) mediates the spread of *bla*_CTX-M-15_ throughout the *Salmonella* spp. Table S4 shows that isolates were divided into seven groups based on their relationship with *bla*_CTX-M_ and ISEc9. However, no difference in the MICs of ampicillin, ceftriaxone, and meropenem was found between these groups.

### Phenotypic resistance prediction using R studio

**Fig 5:**
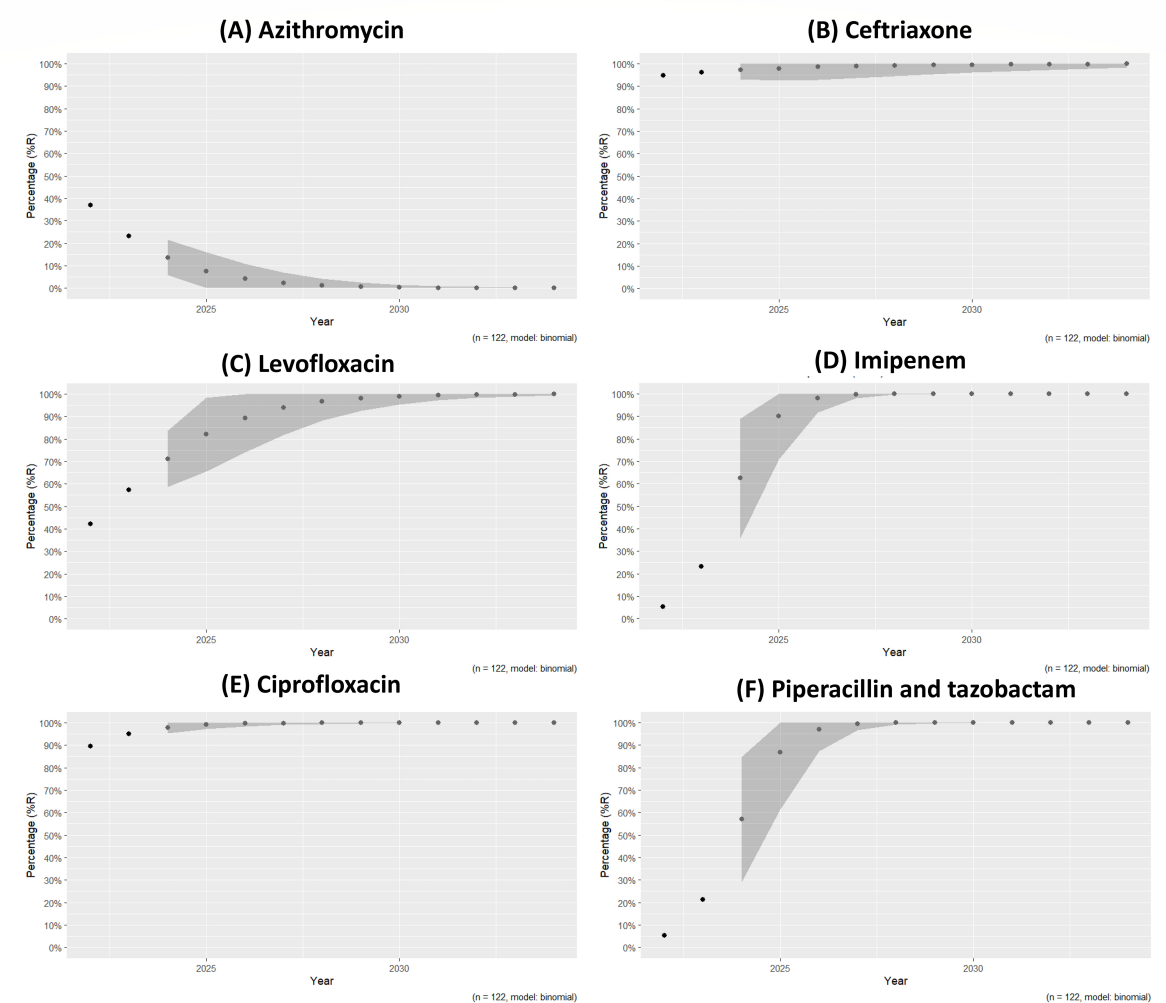
Antibiotic resistance prediction in *S*. Typhi isolates by 2030: A regression model analysis. Prediction model used for major antibiotics (**A**) azithromycin, (**B**) ceftriaxone, (**C**) levofloxacin, (**D**) imipenem, (**E**) ciprofloxacin, and (**F**) piperacillin/tazobactam.

Based on a data column from the phenotypic-resistant data, this model calculates each antibiotic resistance each year and uses a regression model to predict resistance patterns. According to the prediction model, by 2025, antibiotics that are broad-spectrum and empirically used for the treatment of typhoidal infection could push antibiotic resistance up to 100% in cefotaxime, ceftriaxone, ciprofloxacin, cefixime, cefuroxime, and cefuroxime-axetil. Notably, the last line of antibiotics, imipenem, meropenem, levofloxacin, and piperacillin/tazobactam, showed an increased resistance prediction of 100% up to 2030. However, other major antibiotics, such as azithromycin, chloramphenicol, and gentamycin, showed decreased resistance patterns by 2025 (Fig. 5).

### Pangenome analysis

**Fig 6:**
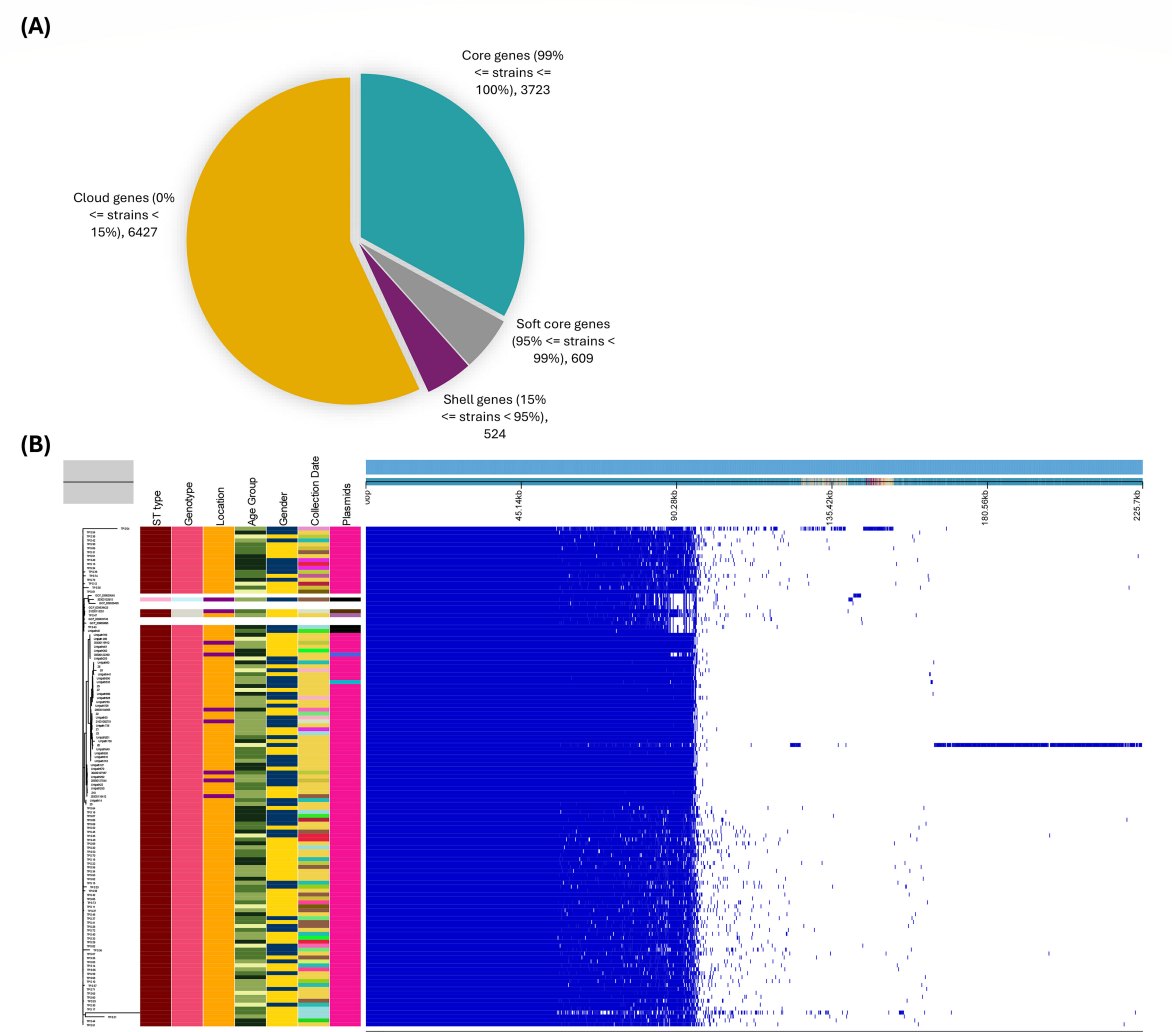
This figure depicts the pangenome analysis of 122 *S*. Typhi isolates from Gujarat, India. (**A**) Number of genes in the pangenome. (**B**) The roary matrix displays gene grouping among 122 *S*. Typhi isolates.

Using a small subset of genomes (*n* = 122), the pangenome distribution between sequence type ST1 and genotype (4.3.1.1 and 4.3.1.2) revealed that 32.99% of genes were shared under the core category, 5.40% under soft core genes, 4.64% under shell genes, and 56.96% under cloud genes (Fig. 6). Most genotypes were grouped into the genotype-specific clade (4.3.2.2), which has been showing an association with the H58 lineage, and the two most prevalent plasmids in the 122 cultures were IncFIB(K) and IncFIB(pHCM2) (Fig. 2 and 6).

## DISCUSSION

Globally, *S*. Typhi is a high-risk pathogen that contributes to increased mortality due to increased MDR to PDR. As treatment becomes increasingly complex, drug-resistant infections, such as those classified as, PDR, XDR, and MDR, rise in death rates (9, 13). In 2024, in an effort to lessen this burden, the World Health Organization (WHO) created the second WHO Bacterial Priority Pathogens List (WHO BPPL), a list of priority pathogens that would encourage research into the creation of new medicines. Depending on the urgency of the need for new antibiotics, pathogens are categorized as critical, high, and medium priority. The FQ-resistant *S*. Typhi has been placed on a high-priority list with an increase in the 11 upper steps globally. The recent rise in resistance to cephalosporins and quinolones in *S*. Typhi can be attributable to an assortment of mechanisms that rapidly formed the resistance pattern (13). This condition limits medical professional treatment with the routinely recommended antibiotics for typhoid fever, particularly ciprofloxacin, azithromycin, and ceftriaxone (14). During the latest 2016 outbreak in Pakistan, approximately 5,000 people became ill because of XDR typhoid fever. Surprisingly, every antibiotic used to treat typhoid fever was shown to harbor *S*. Typhi as a pathogen of XDR (15). A study by Manesh et al. (3) on typhoid and paratyphoid fever reported that 10% of cases of acute febrile illnesses result in hospital visits in India; in total, 80% of all enteric fever cases are attributed to *S*. Typhi. Shetty et al. (4) reported substantial resistance to fluoroquinolones in *S*. Typhi and Paratyphi from tertiary care facilities in coastal Karnataka, India, and suggested third-generation cephalosporins for empirical treatment. A research study in North India found a 5 kb IS91*Sbo1* gene cassette carrying *bla*_CTX-M-15_, *sul2*, *dfrA14*, and *qnrS* genes, which renders bacteria resistant to penicillin, cephalosporin, sulfonamide, and fluoroquinolone in *S*. Typhi isolates (8). The widespread outbreak of *S*. Paratyphi A in Vadodara, India, in 2018 was linked to a point mutation in *gyrA*, associated with reduced susceptibility to fluoroquinolone antibiotics. Specifically, 74.35% of the isolates exhibited an S83F substitution, whereas the remaining showed an S83Y substitution (16). A recent study identified an endemic clone acquiring the IncFIB(K) plasmid, which carries the *bla*_CTX-M-15_ gene in *S*. Typhi strains isolated from Gujarat fall into a unique subclade, specifically genotype 4.3.1.2.2, which is part of the larger genotype 4.3.1.2 (H58 lineage II) that confers resistance to the major class of antibiotics (17).

Considering the significance of typhoidal fever in humans, this investigation examined 122 *S*. Typhi isolated from patients with EF in the Ahmedabad and Vadodara region, Gujarat, India. Most of the *S*. Typhi cases were moderately high in males. A plausible explanation for this finding might be that males are more likely than females to be exposed to the outdoors and eat street food, which might explain this male preponderance. Similarly, Sharvani et al. (18) indicated that the male prevalence of *S*. typhi might be attributed to higher levels of outside exposure. A WHO report states that in the Southeast Asian Region (SEAR), food-borne diseases (FBDs) result in approximately 12 million disability-adjusted living years (DALYs), 150 million illnesses, and 175,000 deaths annually. Approximately one-third (30%) of children under the age of five become ill, and 50,000 die in this region on an annual basis (10, 19). Our analysis found that children under the age of 5 comprise 24.59%, whereas those aged 6–10 comprise 27.80%. This age distribution study demonstrated that *S*. Typhi exposure is more common in children and can be fatal if treatment is limited (20). Furthermore, Mohanty et al. (21) showed a similar pattern of infection among the young population in India.

Antibiotics lower typhoid fever’s morbidity and mortality; however, selecting an antibiotic has become more difficult because of widespread resistance to conventional therapeutic agents. However, the recommended therapy course for susceptible *S*. Typhi infections is still ciprofloxacin (13, 22). Ciprofloxacin is not effective against most *S*. Typhi infections reported in India (22). Ciprofloxacin belongs to the FQs class of drugs that are preferred to treat antibiotic resistance in *S*. Typhi. However, the over-exploitation of FQs for the treatment of typhoid fever has culminated in an increase in *S*. Typhi isolates with reduced susceptibility to FQs (13, 22). Determining the resistance pattern of FQs in the *S*. Typhi isolate is essential for decoding the resistance mechanism. In this study, we investigated FQs resistance using the disc diffusion method. In the FQs class, pefloxacin had the highest resistance (99.18%), followed by ciprofloxacin (94.26%), and levofloxacin had the lowest resistance (54.91%). Comparable with our findings, Sharma et al. (23) noted that pefloxacin is an effective surrogate marker for identifying *S*. Typhi’s susceptibility to ciprofloxacin and levofloxacin. Typhoid fever is a leading foodborne illness in Southeast Asia and Indian region; an interesting study by Vos et al. (19) demonstrated that tourists traveling from India to South Korea carried the highly ciprofloxacin-resistant *S*. Typhi. The *β*-lactam antibiotics, routinely used for the treatment of gram-positive and gram-negative infections, notably ceftriaxone, have reduced susceptibility to *S*. Typhi in South East Asian Region due to the exploitation of antibiotics, which has lessened the treatment choice and led to increased mortality in hospital-acquired illnesses (10, 24). According to Murray et al. (25), gram-negative bacteria have reduced susceptibility to major classes of antibiotics, such as *β*-lactams and FQs, notably in Sub-Saharan Africa and South Asia, contributing to the high mortality rate. Our findings affirm and extend to these findings with a smaller set of data revealing the highest levels of *β* -lactam antibiotic in cefuroxime (97.54%), cefuroxime-axetil (96.72%), ceftriaxone (95.90%), cefazolin (95.08%), ampicillin/sulbactam (95.90%), cefotaxime (94.26%), and cefixime (94.26%). Similarly, Dahiya et al. (8) found the highest rate of cephalosporin resistance in *S*. Typhi isolated from patients with fever in North India. Thus, cephalosporin resistance might be attributed to horizontal gene transfer of *bla*_CTX-M_. According to Akshay et al. (13), horizontal gene transfer is the leading cause of the prevalent dissemination of resistance in *S*. Typhi. Yu et al. (26) also indicated that the *bla*_CTX-M_ gene, which is frequently found in the IncFIB plasmid, contributes to the dissemination of *β*-lactam resistance. Likewise, in the current investigation, *S*. Typhi harbored 89.39% of the *bla*_CTX-M_ gene and contained more than 95% of the IncFIB plasmid that carried the gene. To affirm our result, del Carmen et al. (27) noted the transmission and maintenance of ESBL-encoding genes, especially *bla*_CTX-M_, that are mediated by IncF-type plasmids and have contributed to the rapid rise in antibiotic resistance in disease-causing Enterobacterales.

An effort to curb the FQs -resistant *S*. Typhi burden, which has risen significantly on the WHO priority list, has prompted international and national health organizations to place *S*. Typhi on the high-priority list to accelerate the investigation of the resistance mechanism that reduces the susceptibility of *S*. Typhi. The FQs resistance might be due to various reasons, but two major reasons that have been noted for the spread of resistance are due to QRDR and PMQR in *S*. Typhi. Various factors can cause FQs resistance; however, QRDR and PMQR have been determined to be key contributors to the development of resistance (13). Our findings indicated resistance to surrogate markers such as pefloxacin (99.18%) and ciprofloxacin (94.2%). Furthermore, the study observed two primary factors: QRDR and PMQR; the occurrence of the PMQR gene, as the *qnrS1* gene, was 94.26%; and QRDR mutations were found in *gyrA* (94.12%), *gryB* (100%), and *parC* (6.56%). Meanwhile, Vaithiyam et al. (22) revealed that *S*. Typhi and other gram-negative isolates in tertiary care hospitals exhibited more than 90% ciprofloxacin resistance. Gram-negative isolates that are resistant to ciprofloxacin are primarily due to mutations in *gyrA* and *parC* genes. Mutations in *gyrA* at codon 83 (Thr83Ile) and *parC* (Ser87Leu) can reduce susceptibility to the FQs class of drugs (28). Notably, in the present investigation, mutations were found at Ser83Phen (91.80%), Ser83Leu (1.64%), and Ser83Tyr (0.82%). In *gyrB*, mutations at Ile253Val (100%) and *parC* (6.56%) resulted in multiple mutations. A study conducted in the South India region, India, revealed that double mutation of *gyrA* (Ser83Phen) and *parC* in *S*. Typhi is associated with FQs resistance (13). We speculate that *gyrA*, *gyrB*, and *parC* mutations may alter the targets of topoisomerase subunits, thereby reducing the efficacy of FQs. To confirm the mutation that can reduce effectiveness, an analytical study by Varughese et al. (29) indicated that the *gyrA* residues at 83-codon are essential for FQs interaction. However, modification of the amino acids in the same region resulted in a lower binding energy than that of the wild-type protein complex, thereby displacing the target sight. Most isolates in our investigation were resistant to ciprofloxacin and pefloxacin. More profound and in-depth, we sought to discover whether any general mechanism was involved in the development of FQs resistance. We identified a significant point mutation in the *mdtk* (98.36%) efflux gene, with amino acid alterations from Thr224Lys. Similar findings were reported by Katiyar et al. (30), who noted that *mdtK* could confer resistance to a diverse set of antibiotics viz acriflavine, FQs, and doxorubicin. This study reported the 100% prevalence of the *mdtk* gene in *S*. Typhi. The resistance to pefloxacin and ciprofloxacin in the present study might be attributable to the *mdtk* efflux gene. Furthermore, the phenotype-genotype concordance was examined, and it was observed to be 91.69%. At the same time, less discordance (8.31%) was observed in the phenotype-genotype. Schwan et al. (31) observed an overall concordance of 96.2% and discordance of 3.8% between genotype and phenotype resistance. Likewise, Casaux et al. (32) found an 83.7% association between the phenotypic and genotypic resistance profiles. Again, Vanstokstraeten et al. (33) noted a similar concordance pattern, ranging from 97% for the overall genotype–phenotype.

In this study, broad-spectrum and last-line antibiotics were shown to be ineffective against typhoidal fever, thereby understanding the importance of antibiotic resistance. Furthermore, we tried to identify the dominant antibiotic-resistant lineage in *S*. Typhi. FQs and third-generation cephalosporin resistance in *S*. Typhi are associated with two H58 sublineages (4.3.1.1 and 4.3.1.2) that originated in South Asia, notably India, and are dispersed globally (34). Notably, in the current investigation, we discovered 97.54% H58 lineage II (ST1: 4.3.1.2) and 1.64% H58 lineage I (ST1: 4.3.1.1) in *S*. Typhi (Table 1). Another study by Katiyar et al. (30) investigated the presence of the H58 lineage in *S*. Typhi. This study highlights the presence of H58 lineage I (4.3.1.1) and H58 lineage II (4.3.1.2), which led to a surge in FQs resistance. In addition, Dahiya et al. (8) noted that H58 II is more prevalent in India, Nepal, and Pakistan. The XDR *S*. Typhi outbreak (2016) in Pakistan has revealed the vulnerability of limited treatment options that cause high mortality. The XDR strains revealed the presence of H58 lineage (4.3.1), resulting in an association with FQs and third-generation cephalosporin resistance (35). Interestingly, an intracontinental study by Wong et al. (12) described the presence of H58 carrying both high-level resistance genes PMQR (*qnrS1*) and QRDR (*gyrA* and *parC*). The study reported the presence of an IncFIB plasmid carrying PMQR gene. Moreover, McMillan et al. (36) demonstrated that the IncF plasmid group (FIA, FII, or FIB) in *S*. Typhi may contain various ARGs, highlighting plasmid group compatibility throughout bacterial species. When compared with earlier studies, the results of our study highlight the dissemination of resistance due to the presence of the IncF group and the H58 lineage, both of which pose a public health concern.

Antibiotics, a major discovery in the early 20^th^ century, have transformed treatment techniques and saved many lives. Pathogens such as *S*. Typhi can quickly acquire antibiotic resistance, whereas never-developing drugs might take years to become effective in the hospital. This instructs humanity not to exploit antibiotics to preserve numerous lives while treating infections that are resistant to drugs. Considering the impact of antibiotic resistance, we conducted a primary study to determine whether highly resistant *S*. Typhi can be treated with a combination of antibiotics. The *β*-lactam antibiotics are widely used in clinical practice because of their efficacy against various bacterial infections. However, over time, bacteria have developed mechanisms to resist the action of these antibiotics, primarily through the production of *β*-lactamases. This enzyme neutralizes antibiotics by hydrolyzing their *β*-lactam ring. To inhibit this enzyme, there are several *β*-lactamase inhibitors (BLI), including viz sulbactam, avibactam, and tazobactam. This BLI binds irreversibly or reversibly to *β*-lactamases, thereby preventing them from hydrolyzing *β*-lactam antibiotics (37). In this investigation, we used a combination of ampicillin/sulbactam, broad-spectrum penicillin, and sulbactam, a *β*-lactamase inhibitor. This combination effectively treats illnesses caused by *β*-lactamase-producing bacteria, including urinary tract infections (UTIs), respiratory tract infections, and bacterial sepsis (37). *S*. Typhi typically shows variable resistance to ampicillin. In our study, the MIC of ≤64 µg/mL suggests that some strains may be susceptible, but resistance can also be encountered, especially in regions where this antibiotic has been used extensively. Another important BL/BLI combination used in the current investigation was ceftazidime/avibactam and ceftazidime/tazobactam: ceftazidime is a third-generation cephalosporin antibiotic; avibactam and tazobactam are BLI. It is used to treat severe infections of the intra-abdominal tract, UTIs (including pyelonephritis), and hospital-acquired pneumonia (including ventilator-associated pneumonia) caused by gram-negative bacteria (38). This study showed that ceftazidime/avibactam and ceftazidime/tazobactam are newer combinations against *S*. Typhi, with MICs of 0.12 µg/mL and <0.5 µg/mL, respectively. Based on this primary evidence, we believe that this therapy can treat XDR. *S*. Typhi can preserve life if limited treatment options are available. Furthermore, we used Cefixime/tazobactam: Cefixime is a third-generation cephalosporin antibiotic, whereas tazobactam inhibits *β*-lactamases. This combination effectively cures infections produced by *β*-lactamase-producing organisms, such as severe intra-abdominal infections, UTIs, and nosocomial pneumonia (39). This combination, as in prior investigations, was effective against *S*. Typhi, exhibiting a low MIC of ≤0.5 µg/mL. We also investigated the combination of ceftriaxone/tazobactam and ceftriaxone/sulbactam, a third-generation cephalosporin with a BLI. This combination effectively treats diseases caused by *β*-lactamase-producing bacteria, such as UTIs, pneumonia, and intra-abdominal infections (40). Ceftriaxone/tazobactam also demonstrated a MIC of <0.5 µg/mL, suggesting susceptibility. Ceftriaxone is a crucial antibiotic for treating typhoid fever, although many cases of ceftriaxone resistance have already been reported. The addition of tazobactam enhances its efficacy against *β*-lactamase-producing strains. On the other hand, the MIC of <16 µg/mL for ceftriaxone/sulbactam indicates susceptibility, although some caution may be warranted because resistance can develop due to *β*-lactamase production. The MIC data for *S*. Typhi isolates show susceptibility to rare BL/BLI combinations used to treat EF, such as ceftazidime/avibactam, ceftazidime/tazobactam, cefixime/tazobactam, ceftriaxone/tazobactam, and ceftriaxone/sulbactam. These combinations offer potential therapeutic alternatives, particularly in areas where *S*. Typhi infections are common and resistant to last-line antibiotics.

### Conclusions

Surge of MDR to PDR in *S*. Typhi is a severe public health concern, particularly in South Asia, where the typhoid fever burden is high. Determining FQs and third-generation cephalosporin resistance underscores the urgent need for alternative therapeutic strategies. Our research revealed alarming resistance trends to key antibiotics, such as ceftriaxone, cotrimoxazole, amikacin, ampicillin, cefepime, cefixime, cefotaxime, ciprofloxacin, tetracycline, and gentamicin, limiting effective hospital treatment approaches. Our investigation highlights the genetic pathways underlying FQs resistance, such as point mutations in *gyrA* and *parC*, which lead to treatment failure. The phenotype and genotype analyses revealed an average concordance of 91.69% and a discordance of 8.31%. Notably, in this study, the prevalence of the H58 lineage, mainly H58 lineage I (4.3.1.1) and H58 lineage II (4.3.1.2), was attributed to elevated resistance to third-generation cephalosporins and FQs. This study found the IncFIB plasmid, which carries resistance genes and allows for horizontal gene transfer of *bla*_CTXM_, *qnrS1*, *sul1*, and *dfr14*. This leads to the rapid dissemination of resistance depicted in *S*. Typhi. The machine learning model noted higher 100% resistance prediction for piperacillin/tazobactam, levofloxacin, imipenem, and meropenem up to 2030. In contrast, resistance patterns to gentamycin, azithromycin, and chloramphenicol have declined by 2025. Our findings revealed that combination therapy with *β*-lactam antibiotics and BLI significantly improved the treatment efficacy against XDR *S*. Typhi, contributing to more favorable clinical results and reducing treatment failures. This combination treatment effectively manages infections while avoiding the possibility of additional resistance development. To the best of our knowledge, this is the first report of combination treatment against drug-resistant *S*. Typhi; also, a significant number of H58 lineage II cases have been recorded from the Gujarat region. Nevertheless, further research must be conducted to determine the significance of the H58 lineage in the widespread development of antibiotic resistance; additional confirmation tests are also necessary to validate combination treatment approaches. The current research will contribute to a better understanding of resistance mechanisms and offer different alternatives for managing typhoid fever.

## MATERIALS AND METHODS

### Isolation and MALDI-TOF identification

The study was managed and conducted at the Gujarat Biotechnology Research Centre (GBRC), Government of Gujarat, Gandhinagar, India. These 122 *Salmonella* isolates were collected on a periodic selection (2022–2023) from blood samples collected in the Ahmedabad and Vadodara regions of Gujarat, India, at B.J. Medical College, New Civil Hospital, Ahmedabad, India and Toprani Advanced Lab Systems, Vadodara. The VITEK II compact system (BioMérieux, France) was used to identify the isolates (*n* = 122) and used standard clinical and microbiological techniques. Furthermore, isolates were provided to the GBRC for additional investigation. Matrix-assisted laser desorption ionization (MALDI) with tandem Time-of-Flight (TOF) was employed to validate the *Salmonella* Species. Isolates were grown overnight on non-selective agar plates. A single isolated colony was collected from the agar plate and placed on a MTP 384 spot-polished steel plate. Subsequently, 70% formic acid was added for cell lysis on a steel plate, which allowed it to air-dry. One microliter of α-cyano-4-hydroxycinnamic acid (10 mg/mL) served as a matrix solution prepared in acetonitrile: water:trifluoracetic acid (5:4.75:0.25). Subsequently, samples were examined using an autoflex maX system, MALD-TOF (Bruker Italia S.R.L, Italy). In the linear mode, measurements were conducted over a mass range of 1800–20,000 Da, and data were acquired using flexControl (version 3.4). Every spectrum was imported and cross-referenced using the MALDI Biotyper Compass Explorer (version 4.1.100).

### Screening of antibiotic susceptibility among *Salmonella* isolates

Antibiotic susceptibility testing (AST) was conducted using the disk diffusion method and the VITEK II compact system. AST was initially performed at Toprani Advanced Lab Systems, Vadodara. The findings were reaffirmed by B. J. Medical College, Ahmedabad. AST values were interpreted in accordance with the Clinical and Laboratory Standards Institute (CLSI) document M100 (41). The investigation removed antibiotics that are intrinsically resistant to *Salmonella*. In addition, each batch of testing included *Salmonella enterica* ser. Typhimurium ATCC 14028 as a quality control strain.

### Genomic DNA extraction and whole genome sequencing

The standard method for cetyltrimethylammonium bromide (CTAB), as described by Aboul-Maaty and Oraby (42), was employed with minor modifications to extract genomic DNA (gDNA) from *Salmonella*. The quality of the obtained gDNA was analyzed using agarose gel electrophoresis, and the concentration of gDNA was determined using a Cytation 5 Multimode Reader (Agilent, USA). High-quality gDNA samples of *Salmonella* isolates were processed for WGS. The WGS libraries were prepared using a QIAseq FX DNA Library preparation kit (Qiagen, Germany) according to the manufacturer’s protocol, and paired-end sequencing with 250 × 2 read length was performed using NovaSeq (Illumina, USA) at GBRC.

### Quality control, trimming, assembly, annotation, and pan-genome analysis

The raw sequenced reads were evaluated for quality using FastQC v0.74 (Galaxy Europe) and filtered for low-quality reads and adapter regions using Trimmomatic v.0.36 (43). *De novo* genome assembly was accomplished using SPAdes v3.15.4 (44), and the quality of assembled sequences was assessed using QUAST v5.2.0 (Galaxy Europe). Assembled sequences were annotated using the PATRIC annotation tool v.3.6.2 (45). Raw data were analyzed using FastQC v0.74 (Galaxy Europe). Five *S*. Typhi sequences were downloaded from NCBI and used to compare gene differences with *S*. Typhi WGS. The large-scale prokaryote pan-genome analysis program Roary version 3.12.0 was used to generate the pan-genome of *S*. Typhi from Prokka’s GFF format annotation data. With minor adjustments, the pangenome assembly procedure was performed as described by Page et al. (46) Furthermore, Hadfield et al. (47) described Phandango as an interactive tool used for visualizing gene presence/absence.

### Genomic characterization

The Center of Genomic Epidemiology (CGE database) services were accessed to perform an *in silico* epidemiological analysis of *S*. Typhi (CGE 2024). SeqSerov1.2 was used to predict the serotypes of *Salmonella* isolates for serotype and antigenic profile determination. The species-level identification was performed using ribosomal multi-locus sequence typing (rMLST 2.0), and the assembled genomes were subjected to multi-locus sequence typing using PubMLST (48). Plasmidfinder v2.0.1 and Mobile Element Finder v1.0.3 from the CGE databases used default parameters (95%: minimum identity threshold and 60%: minimum coverage) to study the presence of plasmids. Moreover, chromosomal mutations and acquired antimicrobial resistance were predicted with default parameters using the Resistance Gene Identifier of Comprehensive Antimicrobial Resistance Database 3.2.8 (49). Successively, mutations in ARGs were checked for similarity with reference genes using NCBI’s BLAST. Clustal Omega assessed multiple sequence alignment of ARGs.

### Relationship among susceptibility phenotypes and genotypes of *S*. Typhi

The concordance and discordance analysis resembled the genotypic and phenotypic susceptibility patterns toward the panel of antibiotics and ARGs. All the isolates of *S*. Typhi that were examined using phenotypic antimicrobial susceptibility were correlated with relevant plasmid-mediated ARGs and mutated structural genes. Phenotypically susceptible and intermediate isolates comprise the term “susceptible” in this investigation. The statistical analysis of the genomic method for assessing antibiotic susceptibility was carried out in accordance with Monaghan et al. (50) with minor modifications and tests that included sensitivity, specificity, and positive and negative prediction values.

### Determination of BL/BLI using a microbroth dilution method

The *S*. Typhi isolates that exhibited the highest resistance to a major class of antibiotics were subjected to BL/BLI experiments. As per the CLSI guidelines, the Minimum inhibitory concentrations (MIC) of a group of isolates were determined using the Microbroth dilution (MBD) method (41). All isolates were cultured overnight in Mueller-Hinton broth (MHB) (Himedia, India). Bacterial inoculums were prepared by suspending freshly grown bacteria in normal saline. Furthermore, the inoculum was adjusted to 0.08–0.1 OD at 600 nm (0.5 McFarland standard) using a Multiskan spectrophotometer (Thermo Scientific, USA). The culture was diluted by 1:100 using MHB. Major antibiotics preferred for investigation as per the empirical and cohort treatment suggested for typhoidal fever were ampicillin, ciprofloxacin, co-trimoxazole, chloramphenicol, ceftriaxone, cefixime, imipenem, and pefloxacin. In addition, rare combinations of BL/BLI used for typhoidal fever were examined for the study, including ampicillin/sulbactam, azithromycin, ceftazidime, ceftazidime/avibactam, ceftazidime/tazobactam, cefixime/tazobactam, ceftriaxone/sulbactam, and ceftriaxone/tazobactam. The range of concentration 0.25–128 µg/mL was selected for the study. Subsequently, *S*. Typhi isolates without antibiotics and MHB without inoculum (without antibiotics) were used as growth and negative controls.

### Identification of ceftriaxone resistance genes

From *in silico* analysis, 122 isolates were classified into seven groups, that is, Group 1 (*bla*_CTX-M-15_ associated with ISEc9), Group 2 (*bla*_CTX-M-15_ and ISEc9 not associated), Group 3 (*bla*_CTX-M-194_ and ISEc9 not associated), Group 4 (only *bla*_CTX-M-15_ is present), Group 5 (only ISEc9 is present), Group 6 (both *bla*_CTX-M-15_ and ISEc9 are absent), and WT *S*. Typhi was used as the control. One representative isolate was selected from each group, and the MICs against ampicillin, ceftriaxone, and meropenem using the MBD.

### Phenotypic resistance prediction using R studio

The machine learning model introduced by Berends et al. (51) AMR package for R was performed to predict antimicrobial resistance in *S*. Typhi with the key antibiotic’s azithromycin, chloramphenicol, cefotaxime, cefuroxime, ceftriaxone, ciprofloxacin, cefepime, cefazoline, doxycycline, gentamycin, imipenem, meropenem, levofloxacin, and piperacillin/tazobactam. All the *S*. Typhi isolates (*n* = 122) phenotypic AST data were used to assess future predictions up to 2030 in the development of antibiotic resistance.

## Data Availability

The raw nucleotide sequence reads generated during this investigation have been submitted with the BioProject accession number INRP000326 to the Indian Biological Data Centre (IBDC); the same data are also available with the BioProject accession number PRJEB88981 in the International Nucleotide Sequence Database Collaboration (INSDC) database.

## SUPPLEMENTAL MATERIAL

File S1 spectrum.02540-24-s0001.pdf Bruker MALDI biotyper identification results.

Table S2 spectrum.02540-24-s0002.xlsx Antibiotic sensitivity in *Salmonella*.

Table S3 spectrum.02540-24-s0003.xlsx The details of the isolates and genome assembly statistics.

Table S4 spectrum.02540-24-s0004.docx Distribution of Ceftriaxone-Resistant Genes (blaCTX-M) associated with mobile genetic elements in 122 *S. Typhi* isolates.

ASM does not own the copyrights to Supplemental Material that may be linked to, or accessed through, an article. The authors have granted ASM a non-exclusive, world-wide license to publish the Supplemental Material files. Please contact the corresponding author directly for reuse.

## References

[1] New report calls for urgent action to avert antimicrobial resistance crisis. Available from: https://www.who.int/news/item/29-04-2019-new-report-calls-for-urgent-action-to-avert-antimicrobial-resistance-crisis. Retrieved 09 Sep 2024.

[2] No time to wait: securing the future from drug-resistant infections. Available from: https://www.who.int/publications/i/item/no-time-to-wait-securing-the-future-from-drug-resistant-infections. Retrieved 09 Sep 2024.

[3] Manesh A, Meltzer E, Jin C, Britto C, Deodhar D, Radha S, Schwartz E, Rupali P. 2021. Typhoid and paratyphoid fever: a clinical seminar. J Travel Med 28:taab012. doi:10.1093/jtm/taab012 33550411

[4] Shetty AK, Shetty IN, Furtado ZV, Antony B, Boloor R. 2012. Antibiogram of salmonella isolates from blood with an emphasis on nalidixic acid and chloramphenicol susceptibility in a tertiary care hospital in coastal Karnataka: a prospective study. J Lab Physicians 4:74–77. doi:10.4103/0974-2727.105585 23440906 PMC3574501

[5] Koirala S, Basnyat B, Arjyal A, Shilpakar O, Shrestha K, Shrestha R, Shrestha UM, Agrawal K, Koirala KD, Thapa SD, Karkey A, Dongol S, Giri A, Shakya M, Pathak KR, Campbell J, Baker S, Farrar J, Wolbers M, Dolecek C. 2013. Gatifloxacin versus ofloxacin for the treatment of uncomplicated enteric fever in Nepal: an open-label, randomized, controlled trial. PLoS Negl Trop Dis 7:e2523. doi:10.1371/journal.pntd.0002523 24282626 PMC3837022

[6] Akshay SD, Anupama KP, Deekshit VK, Rohit A, Maiti B. 2022. Effect of sub-minimum inhibitory concentration of ceftriaxone on the expression of outer membrane proteins in Salmonella enterica serovar Typhi. World J Microbiol Biotechnol 38:190. doi:10.1007/s11274-022-03383-5 35972699

[7] Shetty VP, Akshay SD, Rai P, Deekshit VK. 2023. Integrons as the potential targets for combating multidrug resistance in Enterobacteriaceae using CRISPR- Cas9 technique. J Appl Microbiol 134:1–16. doi:10.1093/jambio/lxad137 37410611

[8] Dahiya S, Katiyar A, Rai S, Sharma P, Kapil A, Punit Kaur. 2023. Ceftriaxone-resistant Salmonella Typhi isolated from paediatric patients in north India: insights into genetic profiles and antibiotic resistance mechanisms. Indian J Med Microbiol 46:100448. doi:10.1016/j.ijmmb.2023.100448 37945130

[9] Wong VK, Baker S, Pickard DJ, Parkhill J, Page AJ, Feasey NA, Kingsley RA, Thomson NR, Keane JA, Weill F-X, et al.. 2015. Phylogeographical analysis of the dominant multidrug-resistant H58 clade of Salmonella Typhi identifies inter- and intracontinental transmission events. Nat Genet 47:632–639. doi:10.1038/ng.3281 25961941 PMC4921243

[10] Akshay SD, Deekshit VK, Mohan Raj J, Maiti B. 2023. Outer membrane proteins and efflux pumps mediated multi-drug resistance in Salmonella: rising threat to antimicrobial therapy. ACS Infect Dis 9:2072–2092. doi:10.1021/acsinfecdis.3c00408 37910638

[11] Alikhan N-F, Moreno LZ, Castellanos LR, Chattaway MA, McLauchlin J, Lodge M, O’Grady J, Zamudio R, Doughty E, Petrovska L, Cunha MPV, Knöbl T, Moreno AM, Mather AE. 2022. Dynamics of Salmonella enterica and antimicrobial resistance in the Brazilian poultry industry and global impacts on public health. PLoS Genet 18:e1010174. doi:10.1371/journal.pgen.1010174 35653335 PMC9162342

[12] Wong VK, Baker S, Connor TR, Pickard D, Page AJ, Dave J, Murphy N, Holliman R, Sefton A, Millar M, Dyson ZA, Dougan G, Holt KE, International Typhoid Consortium. 2016. An extended genotyping framework for Salmonella enterica serovar Typhi, the cause of human typhoid. Nat Commun 7:12827. doi:10.1038/ncomms12827 27703135 PMC5059462

[13] Akshay SD, Nayak S, Deekshit VK, Rohit A, Maiti B. 2023. Differential expression of outer membrane proteins and quinolone resistance determining region mutations can lead to ciprofloxacin resistance in Salmonella Typhi. Arch Microbiol 205:1–11. doi:10.1007/s00203-023-03485-0 36961627

[14] Chatterji S, Sharma P, Kaur A, Kalyanasundaram D, Biswas A, Wig N, Kapil A. 2022. A study on the clinical spectrum, prescription pattern and diagnosis of enteric fever in a tertiary care hospital of North India. Int J Community Med Public Health 9:2633. doi:10.18203/2394-6040.ijcmph20221546

[15] Chatham-Stephens K, Medalla F, Hughes M, Appiah GD, Aubert RD, Caidi H, Angelo KM, Walker AT, Hatley N, Masani S, Nash J, Belko J, Ryan ET, Mintz E, Friedman CR. 2019. Emergence of extensively drug-resistant Salmonella typhi infections among travelers to or from Pakistan — United States, 2016–2018. MMWR Morb Mortal Wkly Rep 68:11–13. doi:10.15585/mmwr.mm6801a3 30629573 PMC6342547

[16] Pereira-Dias J, Taneja N, Mahindroo J, Maheshwari G, Patel PJ, Thu TNH, Keane J, Dyson ZA, Baker S, Mylona E. 2023. The genomic characterization of Salmonella Paratyphi a from an outbreak of enteric fever in Vadodara, India. Microb Genom 9:mgen000914. doi:10.1099/mgen.0.000914 36748526 PMC9973848

[17] Priya TT, Jacob JJ, Aravind V, Monisha Priya T, Shah B, Iyer V, Maheshwari G, Trivedi U, Shah A, Patel P, Gaigawale A. 2023. Recent emergence of cephalosporin resistant Salmonella Typhi in India due to the endemic clone acquiring IncFIB(K) plasmid encoding bla_CTX-M-15_ gene. bioRxiv. doi:10.1101/2023.07.05.547856 PMC12054180 40208005

[18] Sharvani R Hemavathi Dayanand DK, Shenoy P, Sarmah P. 2016. Antibiogram of Salmonella Isolates: Time to Consider Antibiotic Salvage. J Clin Diagn Res 10:DC06–8. doi:10.7860/JCDR/2016/18102.7753 PMC4948387 27437211

[19] GBD 2015 Disease and Injury Incidence and Prevalence Collaborators. 2016. Global, regional, and national incidence, prevalence, and years lived with disability for 310 diseases and injuries, 1990–2015: a systematic analysis for the Global Burden of Disease Study 2015. Lancet 388:1545–1602. doi:10.1016/S0140-6736(16)31678-6 27733282 PMC5055577

[20] Sur D, Barkume C, Mukhopadhyay B, Date K, Ganguly NK, Garrett D. 2018. A retrospective review of hospital-based data on enteric fever in India, 2014–2015. J Infect Dis 218:S206–S213. doi:10.1093/infdis/jiy502 30307566 PMC6226629

[21] Mohanty S, Renuka K, Sood S, DAS BK, Kapil A. 2006. Antibiogram pattern and seasonality of Salmonella serotypes in a North Indian tertiary care hospital. Epidemiol Infect 134:961–966. doi:10.1017/S0950268805005844 16476168 PMC2870474

[22] Vaithiyam VS, Rastogi N, Ranjan P, Mahishi N, Kapil A, Dwivedi SN, Soneja M, Wig N, Biswas A, Vaithiyam VS, Rastogi N, Ranjan P, Mahishi N, Kapil A, Dwivedi SN, Soneja M, Wig N, Biswas A. 2020. Antimicrobial resistance patterns in clinically significant isolates from medical wards of a tertiary care hospital in North India. J Lab Physicians 12:196–202. doi:10.1055/s-0040-1721161 33268937 PMC7684999

[23] Sharma P, Dahiya S, Kumari B, Balaji V, Sood S, Das BK, Kapil A. 2017. Pefloxacin as a surrogate marker for quinolone susceptibility in Salmonella enterica serovars Typhi & Paratyphi a in India. Indian J Med Res 145:687–692. doi:10.4103/ijmr.IJMR_494_16 28948961 PMC5644305

[24] Sihombing B, Bhatia R, Srivastava R, Aditama TY, Laxminarayan R, Rijal S. 2023. Response to antimicrobial resistance in South-East Asia region. Lancet Reg Health Southeast Asia 18:100306. doi:10.1016/j.lansea.2023.100306 38028162 PMC10667315

[25] Antimicrobial Resistance Collaborators. 2022. Global burden of bacterial antimicrobial resistance in 2019: a systematic analysis. Lancet 399:629–655. doi:10.1016/S0140-6736(21)02724-0 35065702 PMC8841637

[26] Yu K, Huang Z, Xiao Y, Gao H, Bai X, Wang D. 2024. Global spread characteristics of CTX-M-type extended-spectrum β-lactamases: a genomic epidemiology analysis. Drug Resist Updat 73:101036. doi:10.1016/j.drup.2023.101036 38183874

[27] Rocha-Gracia R del C, Lozano-Zarain P, Gutiérrez Cázarez Z, Alonso CA, Brambila E, Torres C, Cortés-Cortés G. 2022. IncFIB plasmids carrying the resistance gene bla_CTX-M-15_ in ESBL-producing Escherichia coli clones from pediatric patients. J Infect Dev Ctries 16:500–506. doi:10.3855/jidc.15080 35404856

[28] Arabameri N, Heshmatipour Z, Eftekhar Ardebili S, Jafari Bidhendi Z. 2021. The role of gene mutations (gyrA, parC) in resistance to ciprofloxacin in clinical isolates of Pseudomonas aeruginosa. Iran J Pathol 16:426–432. doi:10.30699/IJP.2021.520570.2542 34567192 PMC8463757

[29] Varughese LR, Rajpoot M, Goyal S, Mehra R, Chhokar V, Beniwal V. 2018. Analytical profiling of mutations in quinolone resistance determining region of gyrA gene among UPEC. PLoS One 13:e0190729. doi:10.1371/journal.pone.0190729 29300775 PMC5754135

[30] Katiyar A, Sharma P, Dahiya S, Singh H, Kapil A, Kaur P. 2020. Genomic profiling of antimicrobial resistance genes in clinical isolates of Salmonella Typhi from patients infected with Typhoid fever in India. Sci Rep 10:8299. doi:10.1038/s41598-020-64934-0 32427945 PMC7237477

[31] Schwan CL, Lomonaco S, Bastos LM, Cook PW, Maher J, Trinetta V, Bhullar M, Phebus RK, Gragg S, Kastner J, Vipham JL. 2021. Genotypic and phenotypic characterization of antimicrobial resistance profiles in non-typhoidal Salmonella enterica strains isolated from Cambodian informal markets. Front Microbiol 12:711472. doi:10.3389/fmicb.2021.711472 34603240 PMC8481621

[32] Casaux ML, D’Alessandro B, Vignoli R, Fraga M. 2023. Phenotypic and genotypic survey of antibiotic resistance in Salmonella enterica isolates from dairy farms in Uruguay. Front Vet Sci 10:1055432. doi:10.3389/fvets.2023.1055432 36968467 PMC10033963

[33] Vanstokstraeten R, Piérard D, Crombé F, De Geyter D, Wybo I, Muyldermans A, Seyler L, Caljon B, Janssen T, Demuyser T. 2023. Genotypic resistance determined by whole genome sequencing versus phenotypic resistance in 234 Escherichia coli isolates. Sci Rep 13:449. doi:10.1038/s41598-023-27723-z 36624272 PMC9829913

[34] Carey ME, Thi Nguyen TN, Tran DHN, Dyson ZA, Keane JA, Pham Thanh D, Mylona E, Nair S, Chattaway M, Baker S. 2024. The origins of haplotype 58 (H58) Salmonella enterica serovar Typhi. Commun Biol 7:775. doi:10.1038/s42003-024-06451-8 38942806 PMC11213900

[35] Nizamuddin S, Khan EA, Chattaway MA, Godbole G. 2023. Case of carbapenem-resistant Salmonella Typhi infection, Pakistan, 2022. Emerg Infect Dis 29:2395–2397. doi:10.3201/eid2911.230499 37877663 PMC10617351

[36] McMillan EA, Jackson CR, Frye JG. 2020. Transferable plasmids of Salmonella enterica associated with antibiotic resistance genes. Front Microbiol 11:562181. doi:10.3389/fmicb.2020.562181 33133037 PMC7578388

[37] Ferous S, Anastassopoulou C, Pitiriga V, Vrioni G, Tsakris A. 2024. Antimicrobial and diagnostic stewardship of the novel β-lactam/β-lactamase inhibitors for infections due to carbapenem-resistant Enterobacterales species and Pseudomonas aeruginosa. Antibiotics (Basel) 13:285. doi:10.3390/antibiotics13030285 38534720 PMC10967511

[38] Di Pietrantonio M, Brescini L, Candi J, Gianluca M, Pallotta F, Mazzanti S, Mantini P, Candelaresi B, Olivieri S, Ginevri F, Cesaretti G, Castelletti S, Cocci E, Polo RG, Cerutti E, Simonetti O, Cirioni O, Tavio M, Giacometti A, Barchiesi F. 2022. Ceftazidime–avibactam for the treatment of multidrug-resistant pathogens: a retrospective, single center study. Antibiotics (Basel) 11:321. doi:10.3390/antibiotics11030321 35326784 PMC8944595

[39] McGettigan P, Roderick P, Kadam A, Pollock AMA. 2017. Access, watch, and reserve antibiotics in India: challenges for WHO stewardship. Lancet Glob Health 5:e1075–e1076. doi:10.1016/S2214-109X(17)30365-0 29025629

[40] Shrivastava SM, Saurabh S, Rai D, Dwivedi VK, Chaudhary M. 2009. In vitro microbial efficacy of sulbactomax: a novel fixed dose combination of ceftriaxone sulbactam and ceftriaxone alone. Curr Drug ther 4:73–77. doi:10.2174/157488509787081840

[41] CLSI. 2023. M23 Ed6E. Development of in vitro susceptibility test methods, breakpoints, and quality control parameters. 6th ed

[42] Aboul-Maaty N-F, Oraby H-S. 2019. Extraction of high-quality genomic DNA from different plant orders applying a modified CTAB-based method. Bull Natl Res Cent 43:1–10. doi:10.1186/s42269-019-0066-1

[43] Bolger AM, Lohse M, Usadel B. 2014. Trimmomatic: a flexible trimmer for Illumina sequence data. Bioinformatics 30:2114–2120. doi:10.1093/bioinformatics/btu170 24695404 PMC4103590

[44] Bankevich A, Nurk S, Antipov D, Gurevich AA, Dvorkin M, Kulikov AS, Lesin VM, Nikolenko SI, Pham S, Prjibelski AD, Pyshkin AV, Sirotkin AV, Vyahhi N, Tesler G, Alekseyev MA, Pevzner PA. 2012. SPAdes: a new genome assembly algorithm and its applications to single-cell sequencing. J Comput Biol 19:455–477. doi:10.1089/cmb.2012.0021 22506599 PMC3342519

[45] Wattam AR, Davis JJ, Assaf R, Boisvert S, Brettin T, Bun C, Conrad N, Dietrich EM, Disz T, Gabbard JL, et al.. 2017. Improvements to PATRIC, the all-bacterial Bioinformatics Database and Analysis Resource Center. Nucleic Acids Res 45:D535–D542. doi:10.1093/nar/gkw1017 27899627 PMC5210524

[46] Page AJ, Cummins CA, Hunt M, Wong VK, Reuter S, Holden MTG, Fookes M, Falush D, Keane JA, Parkhill JR. 2015. Roary: rapid large-scale prokaryote pan genome analysis. Bioinformatics 31:3691–3693. doi:10.1093/bioinformatics/btv421 26198102 PMC4817141

[47] Hadfield J, Croucher NJ, Goater RJ, Abudahab K, Aanensen DM, Harris SR. 2018. Phandango: an interactive viewer for bacterial population genomics. Bioinformatics 34:292–293. doi:10.1093/bioinformatics/btx610 29028899 PMC5860215

[48] Jolley KA, Bray JE, Maiden MCJ. 2018. Open-access bacterial population genomics: BIGSdb software, the PubMLST.org website and their applications. Wellcome Open Res 3:124. doi:10.12688/wellcomeopenres.14826.1 30345391 PMC6192448

[49] Alcock BP, Huynh W, Chalil R, Smith KW, Raphenya AR, Wlodarski MA, Edalatmand A, Petkau A, Syed SA, Tsang KK, et al.. 2023. CARD 2023: expanded curation, support for machine learning, and resistome prediction at the comprehensive antibiotic resistance database. Nucleic Acids Res 51:D690–D699. doi:10.1093/nar/gkac920 36263822 PMC9825576

[50] Monaghan TF, Rahman SN, Agudelo CW, Wein AJ, Lazar JM, Everaert K, Dmochowski RR. 2021. Foundational statistical principles in medical research: sensitivity, specificity, positive predictive value, and negative predictive value. Medicina (Kaunas) 57:503. doi:10.3390/medicina57050503 34065637 PMC8156826

[51] Berends MS, Luz CF, Friedrich AW, Sinha BNM, Albers CJ, Glasner CA. 2022. AMR: an R package for working with antimicrobial resistance data. J Stat Softw:104. doi:10.18637/jss.v104.i03

