# Supplementary material for: Comprehensive analysis of extensive drug-resistant Salmonella Typhi in Gujarat region, India: genomic findings and prospective alternative therapy
Source: Microbiol Spectr. 2025 May 27;13(7):e02540-24. doi: 10.1128/spectrum.02540-24 (PMC12211066; doi:10.1128/spectrum.02540-24)
Supplement: File S1 — Bruker MALDI biotyper identification results. [file spectrum.02540-24-s0001.pdf]

Supplementary 1: Bruker MALDI Biotyper Identification Results

Run Info:

**Run Identifier:**	230715-1831-211
**Comment:**	
**Operator:**	Admin@FLEX-PC
**Run Creation Date/Time:**	2023-07-15T19:41:53.099
**Number of Tests:**	75
**Type:**	Standard
**BTS-QC:**	not present
**BTS-QC Position:**	
**Instrument ID:**	1857371.00994
**Server Version:**	4.1.100 (PYTH) 174 2019-06-158_01-16-09

Result Overview

Sample Name	Sample ID	Organism (best match)	Score Value	Organism (second-best match)	Score Value
B15 (+++) (A)	TPS-1 (Standard)	Salmonella sp	2.17	Salmonella sp	2.12
B16 (+) (B)	TPS-2 (Standard)	Salmonella sp	1.89	Salmonella sp	1.85
B17 (+) (B)	TPS-3 (Standard)	Salmonella sp	1.90	Salmonella sp	1.84
B18 (+) (B)	TPS-4 (Standard)	Salmonella sp	1.82	Salmonella sp	1.75
B19 (+) (B)	TPS-5 (Standard)	Salmonella sp	1.88	Salmonella sp	1.87
B20 (+++) (A)	TPS-6 (Standard)	Salmonella sp	2.16	Salmonella sp	2.14
B21 (+++) (A)	TPS-7 (Standard)	Salmonella sp	2.00	Salmonella sp	1.94

Result overview table--continued on next page

Result overview table--continued from previous page

Sample Name	Sample ID	Organism (best match)	Score Value	Organism (second-best match)	Score Value
B22 (+++) (A)	TPS-8 (Standard)	Salmonella sp	2.06	Salmonella sp	1.99
B23 (+) (B)	TPS-9 (Standard)	Citrobacter koseri	1.92	Citrobacter farmeri	1.86
B24 (+) (B)	TPS-10 (Standard)	Salmonella sp	1.72	**No Organism Identification Possible**	1.68
C1 (+) (B)	TPS-11 (Standard)	Salmonella sp	1.90	Salmonella sp	1.85
C2 (+++) (A)	TPS-12 (Standard)	Salmonella sp	2.06	Salmonella sp	1.98
C3 (+) (B)	TPS-13 (Standard)	Salmonella sp	1.92	Salmonella sp	1.91
C4 (+++) (A)	TPS-14 (Standard)	Salmonella sp	2.07	Salmonella sp	1.99
C5 (+) (B)	TPS-15 (Standard)	Salmonella sp	1.86	Salmonella sp	1.86
C6 (+) (B)	TPS-16 (Standard)	Citrobacter koseri	1.86	Citrobacter koseri	1.74
C7 (+) (B)	TPS-17 (Standard)	Salmonella sp	1.93	Salmonella sp	1.90
C8 (+) (C)	TPS-18 (Standard)	Salmonella sp	1.86	Cronobacter sp	1.76
C9 (+) (B)	TPS-19 (Standard)	Salmonella sp	1.81	Salmonella sp	1.76
C10 (+) (B)	TPS-20 (Standard)	Salmonella sp	1.84	Salmonella sp	1.81
C11 (+) (C)	TPS-21 (Standard)	Salmonella sp	1.87	Citrobacter koseri	1.78
C12 (+++) (C)	TPS-22 (Standard)	Salmonella sp	2.04	Citrobacter koseri	1.98
C13 (+++) (A)	TPS-23 (Standard)	Salmonella sp	2.09	Salmonella sp	2.08

Result overview table--continued on next page

Result overview table--continued from previous page

Sample Name	Sample ID	Organism (best match)	Score Value	Organism (second-best match)	Score Value
C14 (+) (B)	TPS-24 (Standard)	Salmonella sp	1.84	Salmonella sp	1.82
C15 (+++) (A)	TPS-25 (Standard)	Salmonella sp	2.20	Salmonella sp	2.17
C16 (+) (C)	TPS-26 (Standard)	Salmonella sp	1.94	Citrobacter koseri	1.90
C17 (+++) (A)	TPS-27 (Standard)	Salmonella sp	2.12	Salmonella sp	2.11
C18 (+) (B)	TPS-28 (Standard)	Salmonella sp	1.99	Salmonella sp	1.91
C19 (+++) (A)	TPS-29 (Standard)	Salmonella sp	2.20	Salmonella sp	1.99
C20 (+) (B)	TPS-30 (Standard)	Salmonella sp	1.95	Salmonella sp	1.89
C21 (+++) (A)	TPS-31 (Standard)	Salmonella sp	2.02	Salmonella sp	2.00
C22 (+) (C)	TPS-32 (Standard)	Salmonella sp	1.94	Citrobacter koseri	1.89
C23 (+) (B)	TPS-33 (Standard)	Salmonella sp	1.95	Salmonella sp	1.92
C24 (+) (B)	TPS-34 (Standard)	Salmonella sp	1.94	Salmonella sp	1.91
D1 (+) (B)	TPS-35 (Standard)	Salmonella sp	1.94	Salmonella sp	1.85
D2 (+) (B)	TPS-36 (Standard)	Salmonella sp	1.97	Salmonella sp	1.90
D3 (+) (B)	TPS-37 (Standard)	Salmonella sp	1.92	Salmonella sp	1.89
D4 (+++) (A)	TPS-38 (Standard)	Salmonella sp	2.02	Salmonella sp	1.89
D5 (+) (B)	TPS-39 (Standard)	Salmonella sp	1.86	Salmonella sp	1.80

Result overview table--continued on next page

Result overview table--continued from previous page

Sample Name	Sample ID	Organism (best match)	Score Value	Organism (second-best match)	Score Value
D6 (+) (B)	TPS-40 (Standard)	Salmonella sp	1.81	Salmonella sp	1.80
D7 (+) (C)	TPS-41 (Standard)	Citrobacter koseri	1.82	Salmonella sp	1.72
D8 (+) (C)	TPS-42 (Standard)	Salmonella sp	1.78	Citrobacter koseri	1.75
D9 (+) (C)	TPS-43 (Standard)	Salmonella sp	1.82	Escherichia coli	1.79
D10 (+) (C)	TPS-44 (Standard)	Salmonella sp	1.76	Enterobacter hormaechei	1.70
D11 (+++) (A)	TPS-45 (Standard)	Salmonella sp	2.17	Salmonella sp	1.98
D12 (+) (B)	TPS-46 (Standard)	Salmonella sp	1.77	Salmonella sp	1.76
D13 (+++) (A)	TPS-47 (Standard)	Salmonella sp	2.16	Salmonella sp	2.16
D14 (+++) (A)	TPS-48 (Standard)	Salmonella sp	2.21	Salmonella sp	2.17
D15 (+) (C)	TPS-49 (Standard)	Salmonella sp	1.83	Citrobacter koseri	1.81
D16 (+) (B)	TPS-50 (Standard)	Salmonella sp	1.98	Salmonella sp	1.96
D17 (+++) (A)	TPS-51 (Standard)	Salmonella sp	2.09	Salmonella sp	2.03
D18 (+) (C)	TPS-52 (Standard)	Salmonella sp	1.76	Citrobacter koseri	1.71
D19 (+) (B)	TPS-61 (Standard)	Salmonella sp	1.89	Salmonella sp	1.86
D20 (+) (B)	TPS-54 (Standard)	Salmonella sp	1.93	Salmonella sp	1.92
D21 (+) (B)	TPS-55 (Standard)	Salmonella sp	1.93	Salmonella sp	1.90

Result overview table--continued on next page

Result overview table--continued from previous page

Sample Name	Sample ID	Organism (best match)	Score Value	Organism (second-best match)	Score Value
D22 (+++) (A)	TPS-56 (Standard)	Salmonella sp	2.01	Salmonella sp	1.90
D23 (+++) (A)	TPS-57 (Standard)	Salmonella sp	2.25	Salmonella sp	2.21
D24 (+) (B)	TPS-58 (Standard)	Salmonella sp	1.81	Salmonella sp	1.71
E1 (+) (B)	TPS-59 (Standard)	Citrobacter koseri	1.88	**No Organism Identification Possible**	1.67
E2 (+) (B)	TPS-60 (Standard)	Salmonella sp	1.73	**No Organism Identification Possible**	1.65
E3 (+) (B)	TPS-62 (Standard)	Salmonella sp	1.93	Salmonella sp	1.88
E4 (+) (B)	TPS-63 (Standard)	Salmonella sp	1.94	Salmonella sp	1.91
E5 (+) (B)	TPS-53 (Standard)	Salmonella sp	1.92	Salmonella sp	1.88
E6 (+) (B)	TPS-64 (Standard)	Salmonella sp	1.88	Salmonella sp	1.85
E7 (+) (B)	TPS-65 (Standard)	Salmonella sp	1.88	Salmonella sp	1.84
E8 (+++) (A)	TPS-66 (Standard)	Salmonella sp	2.00	Salmonella sp	1.96
E9 (+) (B)	TPS-67 (Standard)	Salmonella sp	1.90	Salmonella sp	1.88
E10 (+) (C)	TPS-68 (Standard)	Salmonella sp	1.72	Citrobacter amalonaticus	1.70
E11 (+) (C)	TPS-69 (Standard)	Salmonella sp	1.86	Citrobacter koseri	1.81
E12 (+) (B)	TPS-70 (Standard)	Citrobacter koseri	1.80	**No Organism Identification Possible**	1.58
E13 (-) (C)	TPS-71 (Standard)	**No Organism Identification Possible**	1.69	**No Organism Identification Possible**	1.59

Result overview table--continued on next page

Result overview table--continued from previous page

Sample Name	Sample ID	Organism (best match)	Score Value	Organism (second-best match)	Score Value
E14 (-) (C)	TPS-72 (Standard)	**No Organism Identification Possible**	1.60	**No Organism Identification Possible**	1.51
E15 (+) (B)	TPS-73 (Standard)	Salmonella sp	1.84	Salmonella sp	1.81
E16 (+) (B)	TPS-74 (Standard)	Salmonella sp	1.98	Salmonella sp	1.96
E17 (+++) (C)	TPS-75 (Standard)	Salmonella sp	2.06	Citrobacter koseri	2.02

Bruker MALDI Biotyper Identification Results

Run Info:

**Run Identifier:**	230715-1657-211
**Comment:**	
**Operator:**	Admin@FLEX-PC
**Run Creation Date/Time:**	2023-07-15T17:54:32.731
**Number of Tests:**	28
**Type:**	Standard
**BTS-QC:**	not present
**BTS-QC Position:**	
**Instrument ID:**	1857371.00994
**Server Version:**	4.1.100 (PYTH) 174 2019-06-158_01-16-09

Result Overview

Sample Name	Sample ID	Organism (best match)	Score Value	Organism (second-best match)	Score Value
A11 (+++) (A)	Unipath-690 (Standard)	Salmonella sp	2.07	Salmonella sp	2.01
A12 (+++) (A)	Unipath-828 (Standard)	Salmonella sp	2.11	Salmonella sp	2.11
A13 (+++) (A)	Unipath-549 (Standard)	Salmonella sp	2.15	Salmonella sp	2.09
A14 (+++) (A)	Unipath-153 (Standard)	Salmonella sp	2.15	Salmonella sp	2.13
A15 (+++) (A)	Unipath-646 (Standard)	Salmonella sp	2.01	Salmonella sp	2.00
A16 (+++) (A)	Unipath-22 (Standard)	Salmonella sp	2.12	Salmonella sp	2.12
A17 (+++) (A)	Unipath-750 (Standard)	Salmonella sp	2.27	Salmonella sp	2.22

Result overview table--continued on next page

Result overview table--continued from previous page

Sample Name	Sample ID	Organism (best match)	Score Value	Organism (second-best match)	Score Value
A18 (+++) (A)	Unipath-286 (Standard)	Salmonella sp	2.23	Salmonella sp	2.22
A19 (+++) (A)	Unipath-250 (Standard)	Salmonella sp	2.08	Salmonella sp	2.08
A20 (+++) (A)	Unipath-255 (Standard)	Salmonella sp	2.04	Salmonella sp	2.03
A21 (+++) (A)	Unipath-616 (Standard)	Salmonella sp	2.18	Salmonella sp	2.12
A22 (+++) (A)	Unipath-729 (Standard)	Salmonella sp	2.10	Salmonella sp	2.06
A23 (+++) (A)	Unipath-14 (Standard)	Salmonella sp	2.00	Salmonella sp	2.00
A24 (+++) (A)	Unipath-48 (Standard)	Salmonella sp	2.09	Salmonella sp	2.08
B1 (+++) (A)	Unipath-201 (Standard)	Salmonella sp	2.13	Salmonella sp	2.10
B2 (+) (B)	Unipath-670 (Standard)	Salmonella sp	1.78	Salmonella sp	1.78
B3 (+++) (A)	Unipath-709 (Standard)	Salmonella sp	2.04	Salmonella sp	2.04
B4 (+++) (A)	Unipath-738 (Standard)	Salmonella sp	2.21	Salmonella sp	2.14
B5 (+++) (A)	Unipath-93 (Standard)	Salmonella sp	2.00	Salmonella sp	1.98
B6 (+++) (A)	Unipath-127 (Standard)	Salmonella sp	2.09	Salmonella sp	1.97
B7 (+++) (A)	Unipath-292 (Standard)	Salmonella sp	2.14	Salmonella sp	2.01
B8 (+++) (A)	Unipath-55 (Standard)	Salmonella sp	2.25	Salmonella sp	2.23
B9 (+++) (A)	Unipath-736 (Standard)	Salmonella sp	2.13	Salmonella sp	2.12

Result overview table--continued on next page

Result overview table--continued from previous page					
**Sample Name**	**Sample ID**	**Organism (best match)**	**Score Value**	**Organism (second-best match)**	**Score Value**
B10 (+++) (A)	Unipath-362 (Standard)	Salmonella sp	2.04	Salmonella sp	2.01
B11 (+++) (A)	Unipath-441 (Standard)	Salmonella sp	2.02	Salmonella sp	1.96
B12 (+++) (A)	Unipath-461 (Standard)	Salmonella sp	2.03	Salmonella sp	2.02
B13 (+++) (A)	Unipath-535 (Standard)	Salmonella sp	2.17	Salmonella sp	2.08
B14 (+++) (A)	Unipath-598 (Standard)	Salmonella sp	2.24	Salmonella sp	2.07

Result table for sample 10--continued from previous page			
**Rank (Quality)**	**Matched Pattern**	**Score Value**	**NCBI Identifier**
10 (-)	Citrobacter koseri DSM 46297 DSM	1.35	545

Sample Name	Sample ID	Organism (best match)	Score Value	Organism (second-best match)	Score Value
Result overview table--continued from previous page					
A18 (+++) (A)	Unipath-286 (Standard)	Salmonella sp	2.23	Salmonella sp	2.22
A19 (+++) (A)	Unipath-250 (Standard)	Salmonella sp	2.08	Salmonella sp	2.08
A20 (+++) (A)	Unipath-255 (Standard)	Salmonella sp	2.04	Salmonella sp	2.03
A21 (+++) (A)	Unipath-616 (Standard)	Salmonella sp	2.18	Salmonella sp	2.12
A22 (+++) (A)	Unipath-729 (Standard)	Salmonella sp	2.10	Salmonella sp	2.06
A23 (+++) (A)	Unipath-14 (Standard)	Salmonella sp	2.00	Salmonella sp	2.00
A24 (+++) (A)	Unipath-48 (Standard)	Salmonella sp	2.09	Salmonella sp	2.08
B1 (+++) (A)	Unipath-201 (Standard)	Salmonella sp	2.13	Salmonella sp	2.10
B2 (+) (B)	Unipath-670 (Standard)	Salmonella sp	1.78	Salmonella sp	1.78
B3 (+++) (A)	Unipath-709 (Standard)	Salmonella sp	2.04	Salmonella sp	2.04
B4 (+++) (A)	Unipath-738 (Standard)	Salmonella sp	2.21	Salmonella sp	2.14
B5 (+++) (A)	Unipath-93 (Standard)	Salmonella sp	2.00	Salmonella sp	1.98
B6 (+++) (A)	Unipath-127 (Standard)	Salmonella sp	2.09	Salmonella sp	1.97
B7 (+++) (A)	Unipath-292 (Standard)	Salmonella sp	2.14	Salmonella sp	2.01
B8 (+++) (A)	Unipath-55 (Standard)	Salmonella sp	2.25	Salmonella sp	2.23
B9 (+++) (A)	Unipath-736 (Standard)	Salmonella sp	2.13	Salmonella sp	2.12
Result overview table--continued on next page					

Result overview table--continued from previous page

Sample Name	Sample ID	Organism (best match)	Score Value	Organism (second-best match)	Score Value
B10 (+++) (A)	Unipath-362 (Standard)	Salmonella sp	2.04	Salmonella sp	2.01
B11 (+++) (A)	Unipath-441 (Standard)	Salmonella sp	2.02	Salmonella sp	1.96
B12 (+++) (A)	Unipath-461 (Standard)	Salmonella sp	2.03	Salmonella sp	2.02
B13 (+++) (A)	Unipath-535 (Standard)	Salmonella sp	2.17	Salmonella sp	2.08
B14 (+++) (A)	Unipath-598 (Standard)	Salmonella sp	2.24	Salmonella sp	2.07

Bruker MALDI Biotyper Identification Results

Run Info:

**Run Identifier:**	230715-1656-211
**Comment:**	
**Operator:**	Admin@FLEX-PC
**Run Creation Date/Time:**	2023-07-15T17:42:39.274
**Number of Tests:**	10
**Type:**	Standard
**BTS-QC:**	not present
**BTS-QC Position:**	
**Instrument ID:**	1857371.00994
**Server Version:**	4.1.100 (PYTH) 174 2019-06-158_01-16-09

Result Overview

Sample Name	Sample ID	Organism (best match)	Score Value	Organism (second-best match)	Score Value
A1 (+++) (A)	1-10052023019381 (Standard)	Salmonella sp	2.16	Salmonella sp	2.12
A2 (+) (B)	2-10052022008888 (Standard)	Salmonella sp	1.98	Salmonella sp	1.95
A3 (-) (C)	9-10052022007741 (Standard)	**No Organism Identification Possible**	1.40	**No Organism Identification Possible**	1.38
A4 (+) (B)	3-10052023021149 (Standard)	Salmonella sp	1.80	Salmonella sp	1.73

Result overview table--continued on next page

Result overview table--continued from previous page					
**Sample Name**	**Sample ID**	**Organism (best match)**	**Score Value**	**Organism (second-best match)**	**Score Value**
A5 (+++) (A)	4-10002018147419 (Standard)	Salmonella sp	2.02	Salmonella sp	1.94
A6 (+) (B)	5-10002017094585 (Standard)	Salmonella sp	1.83	Salmonella sp	1.80
A7 (+) (B)	6-10052022000648 (Standard)	Salmonella sp	1.72	**No Organism Identification Possible**	1.66
A8 (-) (C)	7-10052023025358 (Standard)	**No Organism Identification Possible**	1.46	**No Organism Identification Possible**	1.43
A9 (+++) (A)	8-10052023025451 (Standard)	Salmonella sp	2.04	Salmonella sp	2.02
A10 (+) (B)	10-10052023025626 (Standard)	Salmonella sp	1.76	**No Organism Identification Possible**	1.62
