# Supplementary material for: Comprehensive analysis of extensive drug-resistant Salmonella Typhi in Gujarat region, India: genomic findings and prospective alternative therapy
Source: Microbiol Spectr. 2025 May 27;13(7):e02540-24. doi: 10.1128/spectrum.02540-24 (PMC12211066; doi:10.1128/spectrum.02540-24)
Supplement: Table S4 — Distribution of Ceftriaxone-Resistant Genes (blaCTX-M) associated with mobile genetic elements in 122 S. Typhi isolates. [file spectrum.02540-24-s0004.docx]

Table S4: Distribution of Ceftriaxone-Resistant Genes (*bla*_CTX-M_) associated with Mobile Genetic Elements in 122 *S*. Typhi Isolates

| **Groups** | **Ceftriaxone resistant gene** | **Associated MGE** | **List of isolates** | **No. of isolates** |
| --- | --- | --- | --- | --- |
| 1 | *bla_CTX-M-15_* | ISEc9 | 20600104985, 20800127544, 21001002751, 30300118412, 30400119912, 30400107987, 30600122390, TPS01, TPS02, TPS03, TPS04, TPS05, TPS07, TPS08, TPS10, TPS11, RPS13, TPS14, TPS15, TPS16, TPS17, TPS19, TPS20, TPS21, TPS22, TPS23, TPS24, TPS26, TPS27, TPS28, TPS29, TPS30, TPS31, TPS32, TPS33, TPS36, TPS38, TPS39, TPS40, TPS41, TPS42, TPS44, TPS45, TPS46, TPS48, TPS50, TPS52, TPS54, TPS55, TPS56, TPS58, TPS59, TPS60, TPS61, TPS62, TPS65, TPS66, TPS67, TPS68, TPS69, TPS71, TPS73, TPS74, TPS75, Unipath14, Unipath22, Unipath55, Unipath93, Unipath127, Unipath153, Unipath201, Unipath250, Unipath255, Unipath286, Unipath292, Unipath362, Unipath441, Unipath461, Uipath535, Unipath549, Unipath598, Unipath616, Unipath670, Unipath690, Unipath696, Unipath709, Unipath729, Unipath736, Unipath738, Unipath750, Unipath828, Z1, Z2, Z3, Z4, Z5, Z7, Z9, Z10 | 99 |
| 2 | *bla_CTX-M-15_* | ISEc9 | TPS25, TPS37 | 2 |
| 3 | *bla_CTX-M-194_* | ISEc9 | TPS06 | 1 |
| 4 | *bla_CTX-M-15_* | - | TPS09, TPS18, TPS34, TPS35, TPS51, TPS64, Z6, Z8 | 8 |
| 5 | - | ISEc9 | TPS12, TPS53, TPS63, TPS70, TPS72, TPS57 | 6 |
| 6 | - | - | 21000113251, 30300132615, TPS43, TPS47, Unipath48, TPS49 | 6 |
| 7 | - | - | WT | 1 |
